# Supplementary material for: Repurposed inhibitor of bacterial dihydrodipicolinate reductase exhibits effective herbicidal activity
Source: Commun Biol. 2023 May 22;6:550. doi: 10.1038/s42003-023-04895-y (PMC10203105; doi:10.1038/s42003-023-04895-y)
Supplement: Supplementary file 1 — Supplementary Information [file 42003_2023_4895_MOESM1_ESM.pdf]

SUPPLEMENTARY FIGURES

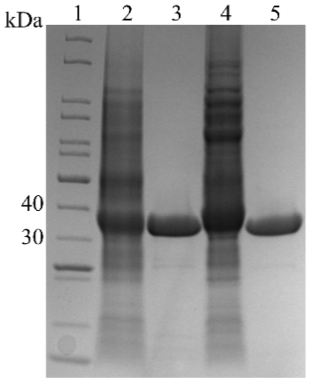

Supplementary Figure 1. Expression and purification of recombinant AtDHDPR enzymes. Lane 1: molecular weight markers (kDa); lane 2: soluble extract of *E. coli* cultures expressing the AtDHDPR1 construct; lane 3: purified recombinant AtDHDPR1; lane 4: soluble extract of *E. coli* cultures expressing the AtDHDPR2 construct; lane 5: purified recombinant AtDHDPR2.

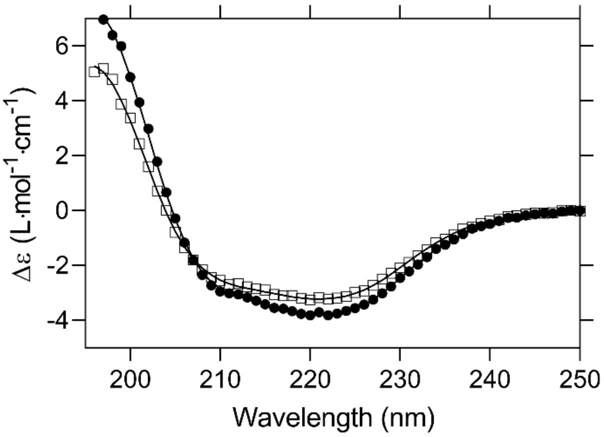

Supplementary Figure 2. Secondary structure of AtDHDPR isoforms. Spectra were collected at a protein concentration of 0.2 mg·mL^{-1} over wavelengths spanning 195-250 nm with a step size of 1.0 nm. The CONTINLL algorithm from the CDPro software package was used to fit the experimental data for AtDHDPR1 (□) and AtDHDPR2 (●) to the SP22X reference set (—). The fit predicted AtDHDPR1 to be comprised of 30% α-helix, 21% β-strand, 12% turn and 37% unordered with a RMSD of 0.061, and AtDHDPR2 to be comprised of 37% α-helix, 22% β-strand, 8% turn and 33% unordered with a RMSD of 0.067.

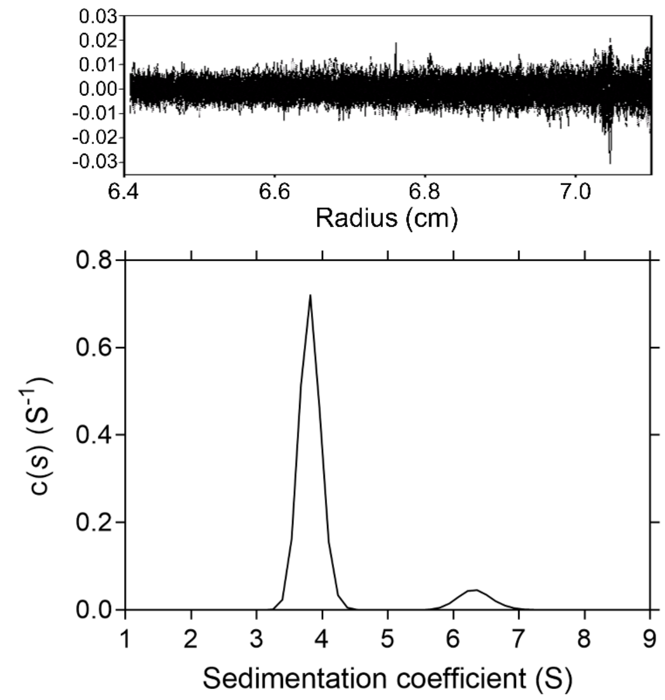

Supplementary Figure 3. Sedimentation velocity analysis by analytical ultracentrifugation of AtDHDPR1. Continuous sedimentation coefficient distribution analysis of AtDHDPR1 at a concentration of 0.9 mg·mL^{-1} resulted in peaks at ~4 S and ~6.5 S, which are consistent with a dimeric species and tetrameric species, respectively. Residuals resulting from nonlinear regression best fits are shown in the top panel.

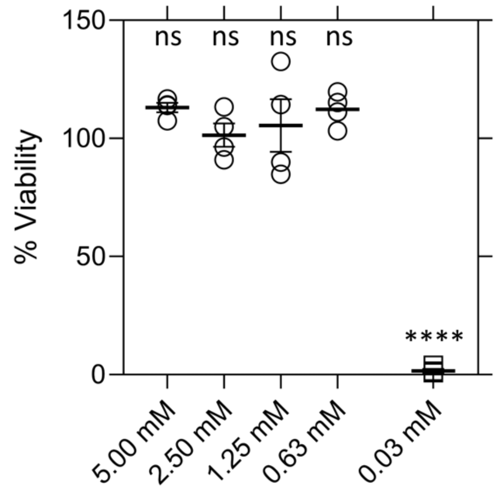

Supplementary Figure 4. Viability of human cells treated with 2,6-PDC. Percentage viability of the HepG2 human cell line after treatment with varying concentrations of 2,6-PDC (circles) or a concentration of the positive control defensin (squares) determined using the MTT assay. Data were normalised against a vehicle control (1% (v/v) DMSO). Data represents mean ± S.E.M. (n = 4). One-way ANOVA multiple comparisons test, ns = not significant, ****P < 0.0001.

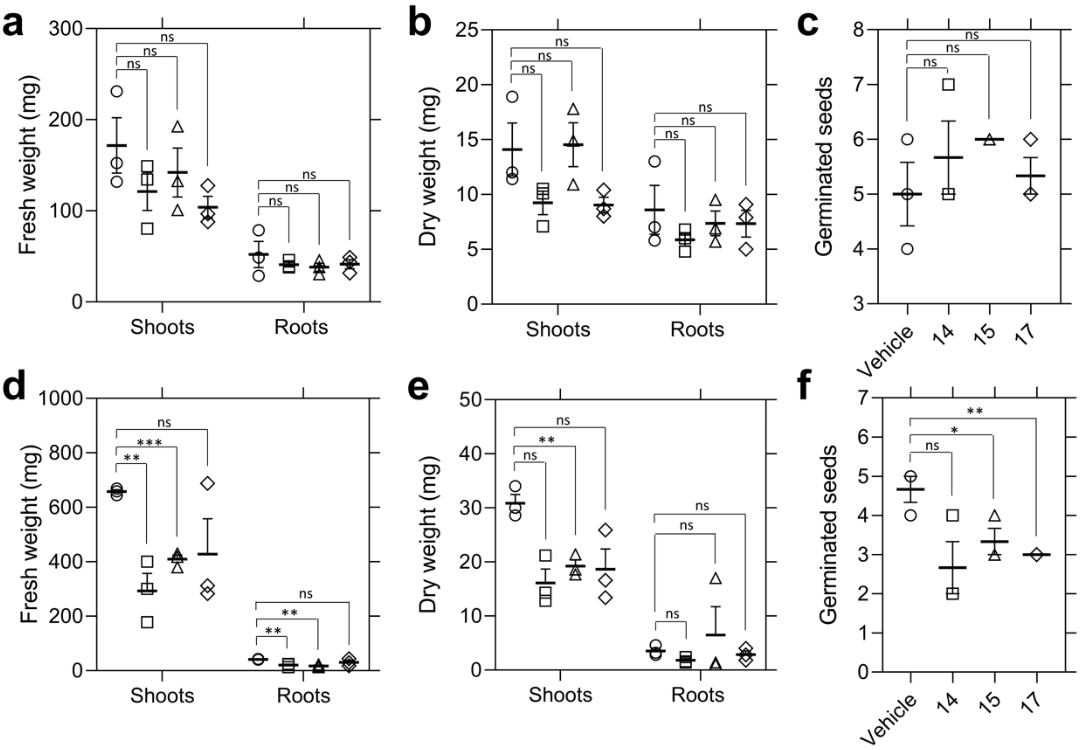

Supplementary Figure 5. Growth and germination of weed species 14 days after treatment with 14, 15 and 17. (a) Fresh and (b) dry weights of *L. rigidum* shoots and roots following treatment of plants with vehicle control (circles) or 1200 mg·L^{-1} of **14** (squares), **15** (triangles) or **17** (diamonds). (c) Number of *L. rigidum* seeds that germinated per pot. (d) Fresh and (e) dry weights of *R. raphanistrum* shoots and roots following treatment of plants with vehicle control (circles) or 1200 mg·L^{-1} of **14** (squares), **15** (triangles) or **17** (diamonds). (f) Number of *R. raphanistrum* seeds that

germinated per pot. Unpaired Student’s two-tailed *t*-test, ns = not significant, *P <0.05, **P <0.01, ***P <0.001. Data represents the mean ± S.E.M shown as lines. (N = 3).

```
                            10         20         30         40         50         60         70         80         90
                    ....|....| ....|....| ....|....| ....|....| ....|....| ....|....| ....|....| ....|....| ....|....|
A. thaliana DHDPR1  MATNGLMA-S SSV--FLHR- --PRIAFASR ---TNQTVGK YGKGRVSFMG IGTRRLPVVL -SMT-AMADS GEEAVK-SVL PGNGISIMVN
A. thaliana DHDPR2  ..A.....-A ...--....P VH.HFS.S.. ---...M.PL GF......I. NVK.CF.... -..G--KSET F...GN-..A ..........
T. cacao DHDPR1      ..SL-.RV-. ANG--.RS-- --EKLPLL.. ---GKRRQ.I A------AKK AAF.WV..AM -.LS-MSTTA IQHNQ.-ATS LDLA.P....
G. max DHDPR2        ...SL.KT-P LN.--.NRHH --NHV..F.N GTSARSSAPI S-------QK RRS.PF...S -MAS-TPVQT SL.KTE-LSS KST.LP....
O. sativa DHDPR1    .LASTFATHP AAAA--AA.- --R.GPIRW. ---LPFCSQI V-------TV TLR..F.MAR L.I.N.L.SQ SL.SAP-AAP .KHSFP.L..
O. sativa DHDPR2    .LSL--RP-P CTL---SP-- ----APWRR. ---RTL---H G-------AA G----T.QRV -.VA--APSA IV.-AV-.PP ARFSFP.L..
H. vulgare DHDPR1   .LSAAFAVHP AT.A--P--- --R.HSVRL. ---QQFCAPI G-------TA TSR.....AM L.I.N.V.SQ SR.SAPA.AP RKLSFP.L..
H. vulgare DHDPR2   .LSV--R.-P PRA--.SP-- ---SPPWRR. ---GRL---D G-------F- A----A.RCV -.AA--PPVT TL.TAT-ARP A.VSFP.L..
P. hallii DHDPR1    .LSATIA.HP AAAAAITL.- --Q.HSLRR. ---QPLR.PI G-------AA APR..METAR S.VVN.V.-- -SQG.P-A.P .KLSFP.L..
P. hallii DHDPR2    .LSL--RP-P HTT--.PR-- ---TSPWLRW ---GRL---G G-------A- A----A.HC. -.AA--APAL AP.-TA-AGP GSVSFP.L..

                           100        110        120        130        140        150        160        170        180
                    ....|....| ....|....| ....|....| ....|....| ....|....| ....|....| ....|....| ....|....| ....|....|
A. thaliana DHDPR1  GCSGKMGKAV IKAADSAGVN IVPISFGSAG EDGQRVEVCG KEITVHGPTE REKVLSSVFE KHPELIVVDY TIPSAVNDNA ELYSKVGVPF
A. thaliana DHDPR2  .......... .......... ...T....VE .A..T..... ...L...... .......... .Y........ .......... ...G......
T. cacao DHDPR1      S.T....... ........LH ...V...AEK .S..TI.L.. ...L....S. K.S..A...H EY.N...... .V.AT..... ..........
G. max DHDPR2        A.T....... .N..EA..L. V..V...CEE .S..TFQIG. ..FL....SD ..S..A..LD .Y.N...... ...D...G.. ..........
O. sativa DHDPR1    S.T....... AE..V...LQ L..V..SAIE VPDGK..I.D R..YIRD.S. G.SI.P.IAK DY.DM..... .V.D...A.. ...C.L.L..
O. sativa DHDPR2    ..T....LS. AE.VT.S.LH L.....S.RD TLDRT.R.GH TDVRIY..SA ..D.....ID EF.DVV.... .A.DS..A.. ...C.L.L..
H. vulgare DHDPR1   S.T.....S. AE..VA..LQ L..V..SAIE VPDGKLNI.D RD.HI.D.S. S.SI.R.IAK DY.DM..... .V.D...A.. ...C.L.L..
H. vulgare DHDPR2   ..T....VS. AE..T.R.LH L..V..S.RE NLDKTIQIGD TD.EIY..SA ..D.....ID EF.DVV.... .A.DS..S.. ...C.L....
P. hallii DHDPR1    S.T....... AE..V...LQ L..V..SATE VPDGKLKI.D R..RI.D.S. S..I.P.IM. EY.D...... .V.D...A.. ...C.L.L.V
P. hallii DHDPR2    ..T.N..LS. AE..VRR.LH L..V..S.RE KVKRTIQ.GQ TD.RLY..SA ..D.....AD EF.DV..... .A.DS..S.. ...C.L.L..

                           190        200        210        220        230        240        250        260        270
                    ....|....| ....|....| ....|....| ....|....| ....|....| ....|....| ....|....| ....|....| ....|....|
A. thaliana DHDPR1  VMGTTGGDRN KLYETVEEAK IYAVISPQMG KQVVAFLAAM EIMAEQFPGA FSGYSLDVME SHQASKLDAS GTAKAVISCF QELGVSYDMD
A. thaliana DHDPR2  .........T R..K....S. .......... .......... ...S...... .A....E... .......... .......... .K........
T. cacao DHDPR1      .........D R..K...DSN G......... .......... .......... ......Q.T. ........T. .......... .K....FG..
G. max DHDPR2        .........D L.HK..LDSN .......... .......... .......... ......Q.L. ......V... .......... NK........
O. sativa DHDPR1    .......N.Q L.HK...D.N V......... .......... .......... ....K.E... ....T...I. .......... .K....FNLN
O. sativa DHDPR2    .........Q L..KS.QDSN N..L...... .......... .......... ....H.E.L. ....G...I. ........A. EK.......N
H. vulgare DHDPR1   .........Q L.NK..QD.N V......... .......... ....K..... .A..K.E... ....T...V. .......... .K...AF.LN
H. vulgare DHDPR2   ........QQ L..KS.QDS. N......... .......... .TI....... ....R.E.L. ....G...T. ........A. EK..AVF...
P. hallii DHDPR1    .........Q L.HK..QD.N .......... .......... ....G...D. ....K.E... ....T...V. .......... .K....F...
P. hallii DHDPR2    .......NQQ L..KS.QDS. N..L...... ........I. R.F....... ....H.E.L. ......K.T. ....D..AS. EK...P...N

                           280        290        300        310        320        330        340        350        360
                    ....|....| ....|....| ....|....| ....|....| ....|....| ....|....| ....|....| ....|....| ....|....|
A. thaliana DHDPR1  Q-IQLIRDPK QQVEMVGVPE EHISGHAFHL YHLTSPDETV SFEFQHNVCG RSIYAEGTVD AVLFLAKKIR LKADQRIYNM IDVLREGNMR
A. thaliana DHDPR2  .-........ ..I.V..... ..V....... .......K.. .......... .......... .......... S..EK..... ..........
T. cacao DHDPR1      .-..M..... ..I....... ..LA...... .......K.. .......... .......... .....S..VQ SS.NK..... ..........
G. max DHDPR2        .-.K.....R ..L....... K.L......M .......D.. T......... .......... .........E T.DPK.L... ..........
O. sativa DHDPR1    E-VKQV...Q E.LTL..... ..L......M .......... .......... .......... .A...H...Q SG.NKKL.D. ..........
O. sativa DHDPR2    R-MVK....E ..L....... ...E...... .......DS. .......... ........S. .AM..HR.V. SNDSK...D. ........S.
H. vulgare DHDPR1   E-VN.V...E E.LAI..... ..LG.....N .......... .......... .......... .AM..HT... SG..KKL.D. .N........
H. vulgare DHDPR2   R-MVK....E ..LY...... ...D...... .......DS. .......... ........S. .AI..Y..VQ SMDPK..... .....Q..D.
P. hallii DHDPR1    E-VK.V...E E..NV..... ..LE.....M .......G.. .......I.. .......... .AM..YT..Q SG.SKKL.D. ..........
P. hallii DHDPR2    RQMVK....D ..L......K ...E...... .......DS. .......... .........I. .AI..HR.VQ S.DSK...D. E......Y..
```

Supplementary Figure 6. Sequence alignment of plant DHDPR enzymes. *A. thaliana* DHDPR1 (UniProt ID: O80574) and DHDPR2 (UniProt ID: Q8LB01), *Theobroma cacao* DHDPR1 (UniProt ID: A0A061DK14), *Glycine max* DHDPR2 (UniProt ID: D2DKE9), *Oryza sativa* DHDPR1 (UniProt ID: Q67W29) and DHDPR2 (UniProt ID: Q10P67), *Hordeum vulgare* DHDPR1 (UniProt ID: F2D3R8) and DHDPR2 (UniProt ID: F2DWQ1) and *Panicum hallii* DHDPR1 (UniProt ID: A0A2T7F6F2) and DHDPR2 (UniProt ID: A0A2T7CF59). Residues are numbered in reference to AtDHDPR1. Identical residues are shown as dots (•), similar (≥50%) residues are shaded in grey, gaps are shown as dashes (-). Sequences were aligned using T-Coffee and edited using BioEdit (v 7.0.5.3).

SUPPLEMENTARY TABLES

Supplementary Table 1. Minimum inhibitory concentration (MIC) values of 2,6-PDC against soil bacteria.

Species	MIC (mM)
Enterobacter ludwigii	>5.0
Cedecea davisae	>5.0
Enterobacter cancerogenus	>5.0

Supplementary Table 2. The published maximal expression levels of DHDPR isoforms and commercial herbicide targets determined by global RNA-sequencing of 79 *A. thaliana* organs and developmental stages.[1]

Target	Commercial Herbicide Mode of Action	Maximum reads per gene
Dihydrodipicolinate reductase 1 *At2G44040.1*	-	45
Dihydrodipicolinate reductase 2 *At3G59890.1*	-	18
Acetolactate synthase *At3G48560*	Branched chain amino acid biosynthesis inhibition	251
5-enolpyruvylshikimate-3-phosphate synthase *At1G48860*	Aromatic amino acid biosynthesis inhibition	40
4-hydroxyphenylpyruvate dioxygenase *At1G06570.1*	Tyrosine catabolism inhibition	146

Supplementary Table 3. Physicochemical properties of lead compounds.

	**14**	**15**	**16**	**17**
Molecular mass ($g \cdot mol^{-1}$)	381.0	292.1	243.2	271.3
clogP	1.453	1.174	0.190	1.148
clogS	-3.413	-2.898	-1.972	-2.256
tPSA	64.96	64.96	64.96	64.96
H-bond acceptors	3	3	3	3
H-bond donors	0	0	0	0
Rotatable bonds	8	8	8	10

Supplementary Methods

General Methods

Commercial solvents and reagents were used as supplied. The petroleum ether used refers to the fraction with 40-60 °C boiling point. Unless otherwise stated, all reactions were monitored by TLC on Polygram® SIL/G25 plates and visualized using UV light (254 nm). ^{1}H, ^{13}C and ^{19}F NMR spectra were recorded on either a Bruker Ascend™ 400 (400 MHz) or a Ultrashield™ 500 PLUS (500 MHz) instrument as dilute solutions in the deuterated solvent. All chemical shifts (δ) are reported in parts per million (ppm) with ^{1}H and ^{13}C NMR referenced to solvent signals [^{1}H NMR: $CDCl_3$ (7.27); ^{13}C NMR: $CDCl_3$ (77.16)]. Coupling constants (*J*) are reported in Hertz (Hz) and recorded after averaging. The multiplicity of the ^{1}H NMR signals are designated by one of the following abbreviations: s=singlet, d=doublet, t=triplet, q=quartet, hept=heptet, m=multiplet, br=broad signal. Infra-red spectra as solutions in $CHCl_3$ or with KBr discs, with the peaks recorded as v_{max} (cm^{-1}). HRMS were obtained using an Agilent 6530 accurate-mass Q-TOF LC/MS in electrospray ionisation (ESI) mode. Melting point data was collected using a Gallenkamp melting point apparatus.

Synthesis of amides

General Procedure

To a solution of pyridine-2,6-dicarbonyl dichloride (51.0 mg, 250 µmol, 1.00 eq) in CH_2Cl_2 (4 mL) was added the required amine (4.00 eq) at 0 °C. The solution was warmed room temperature and stirred for 16 h then diluted with CH_2Cl_2 (10 mL) and washed with brine (10 mL). The organic phase was dried over anhydrous $MgSO_4$, filtered and concentrated under reduced pressure. The crude product was purified by silica gel column chromatography to yield the product.

***N*²,*N*²,*N*⁶,*N*⁶-tetraethylpyridine-2,6-dicarboxamide**[2] **(1)**

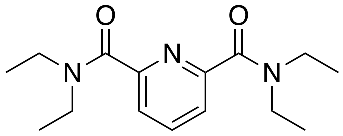

Following general procedure, the crude product was purified by silica gel column chromatography (20% EtOAc in petrol) to yield the title compound (**1**) as yellow solid (42.0 mg, 61%). m.p. 74 °C (lit: 74-77 °C).

^{1}H NMR (500 MHz, $CDCl_3$) δ 7.86 (t, *J* = 7.8 Hz, 1H), 7.61 (d, *J* = 7.8 Hz, 2H), 3.55 (q, *J* = 7.1 Hz, 4H), 3.33 (q, *J* = 7.1 Hz, 4H), 1.25 (t, *J* = 7.1 Hz, 6H), 1.13 (t, *J* = 7.1 Hz, 6H); **^{13}C NMR** (126 MHz, $CDCl_3$) δ 168.2, 153.7, 138.0, 123.7, 43.4, 40.3, 14.3, 12.9; **IR** v_{max} (cm^{-1}): 2988, 1628, 1485, 1217, 752; **HRMS** (ESI) calculated for $C_{15}H_{24}N_3O_2$ $[M+H]^+$ 278.1863, found 278.1864.

***N*^{2}*,N*^{2}*,N*^{6}*,N*^{6}-tetraisopropylpyridine-2,6-dicarboxamide**[3] **(2)**

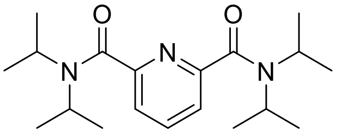

Following general procedure, the crude product was purified by silica gel column chromatography (20% EtOAc in petrol) to yield the title compound (**2**) as a white solid (67.0 mg, 80%). m.p. 161-162 °C (lit: 167 °C).

^{1}H NMR (500 MHz, $CDCl_3$) δ 7.83 (t, *J* = 7.8 Hz, 1H), 7.52 (d, *J* = 7.8 Hz, 2H), 3.89 (hept, *J* = 6.7 Hz, 2H), 3.53 (hept, *J* = 6.8 Hz, 2H), 1.53 (d, *J* = 6.8 Hz, 12H), 1.16 (d, *J* = 6.7 Hz, 12H). **^{13}C NMR** (126 MHz, $CDCl_3$) δ 168.5, 154.8, 138.2, 122.9, 51.1, 46.3, 20.8, 20.6; **IR** ν_{max} (cm^{-1}): 2968, 1634, 1456, 1339, 1040, 772; **HRMS** (ESI) calculated for $C_{19}H_{32}N_3O_2$ $[M+H]^+$ 334.2489, found 334.2498.

Pyridine-2,6-diylbis(morpholinomethanone)[4] **(3)**

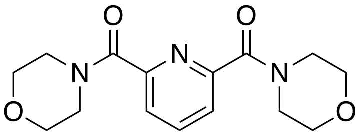

Following general procedure B, the crude product was purified by silica gel column chromatography (30% EtOAc in petrol) to yield the title compound (**3**) as a yellow solid (21.0 mg, 28%). m.p. 120-122 °C (lit: 126 °C).

^{1}H NMR (500 MHz, $CDCl_3$) δ 7.95 (d, *J* = 7.8 Hz, 1H), 7.74 (d, *J* = 7.8 Hz, 2H), 3.87 – 3.78 (m, 8H), 3.67 (dd, *J* = 5.6, 3.7 Hz, 4H), 3.60 (dd, *J* = 5.6, 3.7 Hz, 4H); **^{13}C NMR** (126 MHz, $CDCl_3$) δ 166.8, 152.4, 138.6, 125.2, 67.1, 67.0, 47.9, 43.0; **IR** ν_{max} (cm^{-1}): 2922, 2857, 1636, 1115, 752; **HRMS** (ESI) calculated for $C_{15}H_{20}N_3O_4$ $[M+H]^+$ 306.1448, found 306.1443.

***N*^{2}*,N*^{2}*,N*^{6}*,N*^{6}-tetramethylpyridine-2,6-dicarboxamide**[5] **(4)**

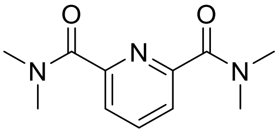

Following general procedure, the crude product was purified by silica gel column chromatography (20% EtOAc in petrol) to yield the title compound (**4**) as a white solid (23.0 mg, 42%). m.p. 144-145°C (lit: 144.5-148 °C).

^{1}H NMR (500 MHz, $CDCl_3$) δ 7.89 (t, *J* = 7.8 Hz, 1H), 7.66 (d, *J* = 7.8 Hz, 2H), 3.14 (s, 6H), 3.05 (s, 6H). **^{13}C NMR** (126 MHz, $CDCl_3$) δ 168.4, 153.3, 138.2, 124.2, 39.2, 35.9;

IR v_{max} (cm^{-1}): 2928, 1634, 1506, 1391, 1103, 1082, 835; **HRMS** (ESI) calculated for $C_{11}H_{16}N_3O_2$ $[M+H]^+$ 222.1237, found 222.1237.

***N*[2],*N*[6]-diethylpyridine-2,6-dicarboxamide**[6] **(5)**

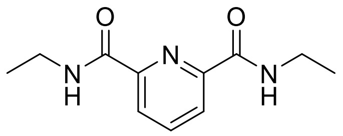

Following general procedure, the crude product was purified by silica gel column chromatography (25% EtOAc in petrol) to yield the title compound (**5**) as a white solid (30.0 mg, 54%). m.p. 178-179 °C (lit: 184.2-184.6 °C).

^{1}H NMR (500 MHz, $CDCl_3$) δ 8.35 (d, *J* = 7.8 Hz, 2H), 8.00 (t, *J* = 7.8 Hz, 1H), 7.92 (s, 2H), 3.52 (m, 4H), 1.26 (m, 6H). **^{13}C NMR** (126 MHz, $CDCl_3$) δ 163.6, 149.1, 139.1, 125.0, 34.6, 15.1; **IR** v_{max} (cm^{-1}): 3308, 2972, 1655, 1533, 1447; **HRMS** (ESI) calculated for $C_{11}H_{16}N_3O_2$ $[M+H]^+$ 222.1237, found 222.1241.

Pyridine-2,6-diylbis(pyrrolidin-1-ylmethanone)[7] **(6)**

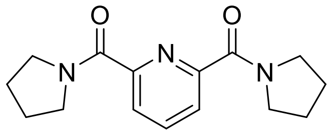

Following general procedure, the crude product was purified by silica gel column chromatography (20% EtOAc in petrol) to yield the title compound (**6**) as a yellow solid (45.0 mg, 66%). m.p. 78-80 °C.

^{1}H NMR (500 MHz, $CDCl_3$) δ 7.92 – 7.82 (m, 3H), 3.66 (m, 8H), 1.97 – 1.86 (m, 8H). **^{13}C NMR** (126 MHz, $CDCl_3$) δ 166.2, 153.1, 137.8, 124.9, 49.2, 47.0, 26.7, 24.1; **IR** v_{max} (cm^{-1}): 2978, 1622, 1456, 1412, 1217, 752; **HRMS** (ESI) calculated for $C_{15}H_{20}N_3O_2$ $[M+H]^+$ 274.1550, found 274.1558.

***N*[2],*N*[6]-Di(prop-2-yn-1-yl)pyridine-2,6-dicarboxamide**[8] **(7)**

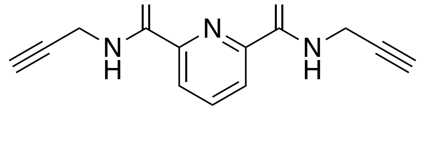

To a solution of pyridine-2,6-dicarbonyl dichloride (51.0 mg, 250 µmol, 1.00 eq) and Et_3N (70.0 µL, 500 µmol, 2.00 eq) was added propargylamine (32.0 µL, 500 µmol, 2.00 eq). The solution was stirred at room temperature for 16 h then diluted with CH_2Cl_2 (10

mL) and washed with brine (10 mL). The organic phase was dried over anhydrous $MgSO_4$, filtered and concentrated under reduced pressure. The crude residue was purified by silica gel column chromatography (35% EtOAc in petrol) to yield the title compound (**7**) as a white solid (37.0 mg, 62%). m.p. 178-180 °C (lit: 188 °C).

^{1}H NMR (500 MHz, $CDCl_3$) δ 8.40 (d, *J* = 7.8 Hz, 2H), 8.06 (t, *J* = 7.8 Hz, 1H), 7.93 (s, 2H), 4.34 (dd, *J* = 5.7, 2.5 Hz, 4H), 2.33 – 2.28 (m, 2H); **^{13}C NMR** (126 MHz, $CDCl_3$) δ 163.3, 148.6, 139.3, 125.7, 79.4, 72.0, 29.4; **IR** ν_{max} (cm^{-1}): 3294, 1661, 1522, 1445; **HRMS** (ESI) calculated for $C_{13}H_{11}N_3NaO_2$ $[M+Na]^+$ 264.0743, found 264.0747.

Synthesis of Esters

Dimethyl pyridine-2,6-dicarboxylate[9] (8)

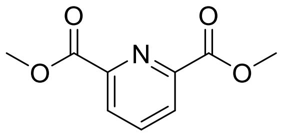

To a solution of pyridine-2,6-dicarbonyl dichloride (408 mg, 2.00 mmol) in CH_2Cl_2 (10 mL) was added MeOH (243 µL, 6.00 mmol) followed by Et_3N (1.12 mL, 8.00 mmol). The solution was stirred at room temperature for 2 h, diluted with CH_2Cl_2 (50 mL) and washed with water (50 mL). The organic phase was dried over anhydrous $MgSO_4$, filtered and concentrated under reduced pressure. The crude residue was purified by silica gel column chromatography (0-40% EtOAc in petrol) to yield the title compound (**8**) as an off-white solid (281 mg, 72%). m.p. 121-122 °C (lit: 120-122 °C)

^{1}H NMR (500 MHz, $CDCl_3$) δ 8.32 (d, *J* = 7.8 Hz, 2H), 8.03 (t, *J* = 7.8 Hz, 1H), 4.03 (s, 6H). **^{13}C NMR** (126 MHz, $CDCl_3$) δ 165.2, 148.4, 138.5, 128.2, 53.3; **IR** ν_{max} (cm^{-1}): 1751, 1715, 1450, 1323, 1290, 1252, 1144, 739; **HRMS** (ESI) calculated for $C_9H_{10}NO_4$ $[M+H]^+$ 196.0604, found 196.0613.

Diethyl pyridine-2,6-dicarboxylate[10] (9)

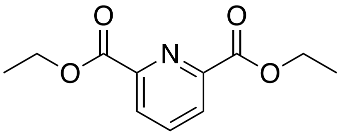

To a solution of pyridine-2,6-dicarbonyl dichloride (408 mg, 2.00 mmol) in CH_2Cl_2 (10 mL) was added EtOH (350 µL, 6.00 mmol) followed by Et_3N (1.12 mL, 8.00 mmol). The solution was stirred at room temperature for 1 h, diluted with CH_2Cl_2 (50 mL) and washed with water (50 mL). The organic phase was dried over anhydrous $MgSO_4$, filtered and concentrated under reduced pressure. The crude residue was purified by silica gel column chromatography (0-30% EtOAc in petrol) to yield the title compound (**9**) as an off-white solid (273 mg, 61%). m.p. 43-45 °C (lit: 45-46.5 °C).

^{1}H NMR (500 MHz, $CDCl_3$) δ 8.27 (d, *J* = 7.8 Hz, 2H), 7.99 (t, *J* = 7.8 Hz, 1H), 4.48 (q, *J* = 7.2 Hz, 4H), 1.45 (t, *J* = 7.2 Hz, 6H); **^{13}C NMR** (126 MHz, $CDCl_3$) δ 164.7, 148.7, 138.3, 127.9, 62.4, 14.3. **IR** ν_{max} (cm^{-1}): 1744, 1719, 1369, 1321, 1242, 1138, 1024, 752; **HRMS** (ESI) calculated for $C_{11}H_{14}NO_4$ $[M+H]^+$ 224.0917, found 224.0919.

Dipropyl pyridine-2,6-dicarboxylate[11] **(10)**

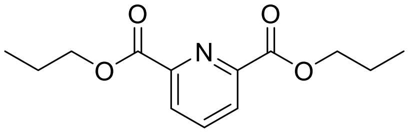

To a solution of pyridine-2,6-dicarbonyl dichloride (240 mg, 1.18 mmol) in CH_2Cl_2 (10 mL) was added n-propanol (264 µL, 3.53 mmol) followed by Et_3N (658 µL, 4.72 mmol). The solution was stirred at room temperature for 2 h, diluted with CH_2Cl_2 (50 mL) and washed with water (50 mL). The organic phase was dried over anhydrous $MgSO_4$, filtered and concentrated under reduced pressure. The crude residue was purified by silica gel column chromatography (0-30% EtOAc in petrol) to yield the title compound (**10**) as a pale yellow oil (162 mg, 55%).

^{1}H NMR (400 MHz, $CDCl_3$) δ 8.27 (d, *J* = 7.8 Hz, 2H), 7.99 (t, *J* = 7.8 Hz, 1H), 4.38 (t, *J* = 6.9 Hz, 4H), 1.95 – 1.72 (m, 4H), 1.05 (t, *J* = 7.4 Hz, 6H). **^{13}C NMR** (101 MHz, $CDCl_3$) δ 164.8, 148.8, 138.3, 127.8, 67.9, 22.1, 10.5; **IR** ν_{max} (cm^{-1}): 2968, 1748, 1719, 1323, 1240, 1165, 1140, 752; **HRMS** (ESI) calculated for $C_{13}H_{18}NO_4$ $[M+H]^+$ 252.1230, found 252.1236.

Dibutyl pyridine-2,6-dicarboxylate[12] **(11)**

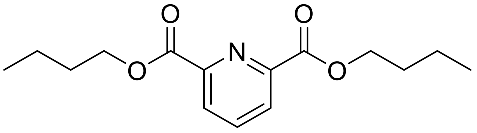

To a solution of pyridine-2,6-dicarbonyl dichloride (408 mg, 2.00 mmol) in CH_2Cl_2 (10 mL) was added n-butanol (550 µL, 6.00 mmol) followed by Et_3N (1.12 mL, 8.00 mmol). The solution was stirred at room temperature for 2 h, diluted with CH_2Cl_2 (50 mL) and washed with water (50 mL). The organic phase was dried over anhydrous $MgSO_4$, filtered and concentrated under reduced pressure. The crude residue was purified by silica gel column chromatography (0-30% EtOAc in petrol) to yield the title compound (**11**) as an off-white solid (405 mg, 73%). m.p. 62-64 °C (lit: 66-67 °C).

^{1}H NMR (400 MHz, $CDCl_3$) δ 8.26 (d, *J* = 7.8 Hz, 2H), 7.99 (t, *J* = 7.8 Hz, 1H), 4.43 (t, *J* = 6.8 Hz, 4H), 1.87 – 1.75 (m, 4H), 1.54 – 1.43 (m, 4H), 0.99 (t, *J* = 7.4 Hz, 6H). **^{13}C NMR** (101 MHz, $CDCl_3$) δ 164.8, 148.9, 138.3, 127.8, 66.2, 30.8, 19.3, 13.9; **IR** ν_{max}

(cm^{-1}): 2955, 1740, 1578, 1290, 1248, 766; **HRMS** (ESI) calculated for $C_{15}H_{22}NO_4$ $[M+H]^+$ 280.1543, found 280.1548.

Dipentyl pyridine-2,6-dicarboxylate[13] (12)

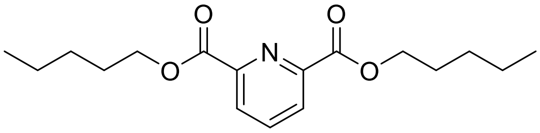

To a solution of pyridine-2,6-dicarbonyl dichloride (550 mg, 2.70 mmol) in CH_2Cl_2 (20 mL) was added n-pentanol (877 µL, 8.10 mmol) followed by Et_3N (1.51 mL, 10.8 mmol). The solution was stirred at room temperature for 2 h, diluted with CH_2Cl_2 (50 mL) and washed with water (50 mL). The organic phase was dried over anhydrous $MgSO_4$, filtered and concentrated under reduced pressure. The crude residue was purified by silica gel column chromatography (0-30% EtOAc in petrol) to yield the title compound (**12**) as an off-white solid (340 mg, 41%). m.p. 39-41 °C.

^{1}H NMR (500 MHz, $CDCl_3$) δ 8.27 (d, *J* = 7.8 Hz, 2H), 8.00 (t, *J* = 7.8 Hz, 1H), 4.42 (t, *J* = 6.9 Hz, 4H), 1.84 (dd, *J* = 8.1, 6.9 Hz, 4H), 1.49 – 1.34 (m, 8H), 0.93 (t, *J* = 7.1 Hz, 6H); **^{13}C NMR** (126 MHz, $CDCl_3$) δ 164.8, 148.9, 138.2, 127.8, 66.5, 28.4, 28.2, 22.5, 14.1; **IR** v_{max} (cm^{-1}): 2957, 1741, 1734, 1719, 1325, 1240, 1144; **HRMS** (ESI) calculated for $C_{17}H_{26}NO_4$ $[M+H]^+$ 308.1856, found 308.1866.

Dihexyl pyridine-2,6-dicarboxylate[14] (13)

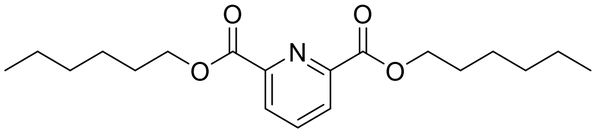

To a solution of pyridine-2,6-dicarbonyl dichloride (550 mg, 2.70 mmol) in CH_2Cl_2 (20 mL) was added n-hexanol (1.02 mL, 8.10 mmol) followed by Et_3N (1.51 mL, 10.8 mmol). The solution was stirred at room temperature for 2 h, diluted with CH_2Cl_2 (50 mL) and washed with water (50 mL). The organic phase was dried over anhydrous $MgSO_4$, filtered and concentrated under reduced pressure. The crude residue was purified by silica gel column chromatography (0-30% EtOAc in petrol) to yield the title compound (**13**) as a low melting colourless solid (608 mg, 67%).

^{1}H NMR (400 MHz, $CDCl_3$) δ 8.25 (d, *J* = 7.8 Hz, 2H), 7.99 (t, *J* = 7.8 Hz, 1H), 4.40 (t, *J* = 7.0 Hz, 4H), 1.88 – 1.76 (m, 4H), 1.49 – 1.39 (m, 4H), 1.39 – 1.26 (m, 8H), 0.93 – 0.85 (m, 6H); **^{13}C NMR** (101 MHz, $CDCl_3$) δ 164.8, 148.8, 138.2, 127.8, 66.5, 31.6, 28.6, 25.7, 22.6, 14.1; **IR** v_{max} (cm^{-1}): 2955, 1740, 1576, 1290, 1250, 764; **HRMS** (ESI) calculated for $C_{19}H_{30}NO_4$ $[M+H]^+$ 336.2169, found 336.2162.

Bis(2-bromoethyl) pyridine-2,6-dicarboxylate (14)

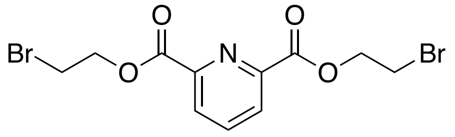

To a solution of pyridine-2,6-dicarbonyl dichloride (550 mg, 2.70 mmol) in CH_2Cl_2 (20 mL) was added 2-bromoethanol (574 µL, 8.10 mmol) followed by Et_3N (1.51 mL, 10.8 mmol). The solution was stirred at room temperature for 2 h, diluted with CH_2Cl_2 (50 mL) and washed with water (50 mL). The organic phase was dried over anhydrous $MgSO_4$, filtered and concentrated under reduced pressure. The crude residue was purified by silica gel column chromatography (10-50% EtOAc in petrol) to yield the title compound (**14**) as an off-white solid (532 mg, 52%). m.p. 104-106 °C.

^{1}H NMR (400 MHz, $CDCl_3$) δ 8.33 (d, *J* = 7.8 Hz, 2H), 8.05 (t, *J* = 7.8 Hz, 1H), 4.73 (t, *J* = 6.5 Hz, 4H), 3.70 (t, *J* = 6.5 Hz, 4H); **^{13}C NMR** (101 MHz, $CDCl_3$) δ 164.0, 148.2, 138.6, 128.5, 65.3, 28.1; **IR** ν_{max} (cm^{-1}): 2924, 1748, 1724, 1379, 1317, 1242, 1142, 750; **HRMS** (ESI) calculated for $C_{11}H_{11}Br_2NNaO_4$ $[M+Na]^+$ 401.8947, found 401.8944.

Bis(2-chloroethyl) pyridine-2,6-dicarboxylate[15] (15)

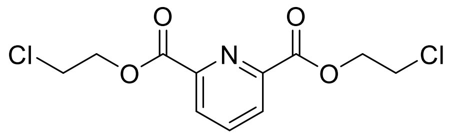

To a solution of pyridine-2,6-dicarbonyl dichloride (408 mg, 2.00 mmol) in CH_2Cl_2 (20 mL) was added 2-chloroethanol (402 µL, 6.00 mmol) followed by Et_3N (1.12 mL, 8.00 mmol). The solution was stirred at room temperature for 2 h, diluted with CH_2Cl_2 (50 mL) and washed with water (50 mL). The organic phase was dried over anhydrous $MgSO_4$, filtered and concentrated under reduced pressure. The crude residue was purified by silica gel column chromatography (0-30% EtOAc in petrol) to yield the title compound (**15**) as an off-white solid (342 mg, 58%). m.p. 94-96 °C (lit: 96 °C).

^{1}H NMR (400 MHz, $CDCl_3$) δ 8.33 (d, *J* = 7.8 Hz, 2H), 8.05 (t, *J* = 7.8 Hz, 1H), 4.68 (t, *J* = 6.0 Hz, 4H), 3.87 (t, *J* = 6.0 Hz, 4H). **^{13}C NMR** (101 MHz, $CDCl_3$) δ 164.1, 148.2, 138.6, 128.5, 65.5, 41.2; **IR** ν_{max} (cm^{-1}): 2957, 1749, 1719, 1319, 1236, 1142, 750; **HRMS** (ESI) calculated for $C_{11}H_{12}Cl_2NO_4$ $[M+H]^+$ 292.0138, found 292.0132.

Di(prop-2-yn-1-yl) pyridine-2,6-dicarboxylate[16] (16)

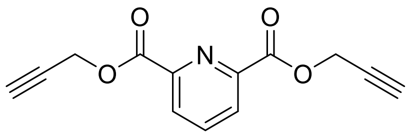

To a solution of pyridine-2,6-dicarbonyl dichloride (550 mg, 2.70 mmol) in CH_2Cl_2 (20 mL) was added propargyl alcohol (466 µL, 8.10 mmol) followed by Et_3N (1.50 mL, 10.8 mmol). The solution was stirred at room temperature for 2 h, diluted with CH_2Cl_2 (50 mL) and washed with water (50 mL). The organic phase was dried over anhydrous $MgSO_4$, filtered and concentrated under reduced pressure to yield the title compound (**16**) as an off-white solid (562 mg, 86%). m.p. 123-124 °C.

^{1}H NMR (500 MHz, $CDCl_3$) δ 8.34 (d, *J* = 7.8 Hz, 2H), 8.05 (t, *J* = 7.8 Hz 1H), 5.02 (d, *J* = 2.5 Hz, 4H), 2.54 (t, *J* = 2.5 Hz, 2H). **^{13}C NMR** (126 MHz, $CDCl_3$) δ 163.8, 148.0, 138.6, 128.6, 77.2, 75.8, 53.7; **IR** ν_{max} (cm^{-1}): 3258, 1732, 1315, 1134, 1121, 1078; **HRMS** (ESI) calculated for $C_{13}H_{10}NO_4$ $[M+H]^+$ 244.0604, found 244.0609.

Di(but-3-yn-1-yl) pyridine-2,6-dicarboxylate (17)

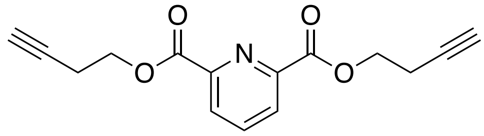

To a solution of pyridine-2,6-dicarbonyl dichloride (408 mg, 2.00 mmol) in CH_2Cl_2 (20 mL) was added 3-butyn-1-ol (454 µL, 6.00 mmol) followed by Et_3N (1.12 mL, 8.00 mmol). The solution was stirred at room temperature for 1 h, diluted with CH_2Cl_2 (50 mL) and washed with water (50 mL). The organic phase was dried over anhydrous $MgSO_4$, filtered and concentrated under reduced pressure. The crude residue was purified by silica gel column chromatography (0-30% EtOAc in petrol) to yield the title compound (**17**) as an off-white solid (415 mg, 77%). m.p. 86-88 °C.

^{1}H NMR (500 MHz, $CDCl_3$) δ 8.29 (d, *J* = 7.8 Hz, 2H), 8.02 (t, *J* = 7.8 Hz, 1H), 4.54 (t, *J* = 7.2 Hz, 4H), 2.74 (td, *J* = 7.2, 2.7 Hz, 4H), 2.04 (t, *J* = 2.7 Hz, 2H); **^{13}C NMR** (126 MHz, $CDCl_3$) δ 164.3, 148.4, 138.4, 128.2, 79.7, 70.5, 63.8, 19.1; **IR** ν_{max} (cm^{-1}): 3289, 2959, 1748, 1717, 1325, 1240, 1144, 1084, 752; **HRMS** (ESI) calculated for $C_{15}H_{14}NO_4$ $[M+H]^+$ 272.0917, found 272.0926.

Bis(2,2,2-trifluoroethyl) pyridine-2,6-dicarboxylate[15] (18)

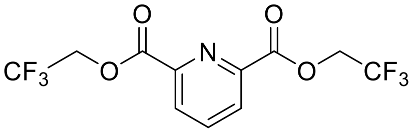

To a solution of pyridine-2,6-dicarbonyl dichloride (408 mg, 2.00 mmol) in CH_2Cl_2 (20 mL) was added 2,2,2-trifluoroethanol (432 µL, 6.00 mmol) followed by Et_3N (1.12 mL, 8.00 mmol). The solution was stirred at room temperature for 2 h, diluted with CH_2Cl_2 (50 mL) and washed with water (50 mL). The organic phase was dried over anhydrous $MgSO_4$, filtered and concentrated under reduced pressure. The crude residue was purified by silica gel column chromatography (0-30% EtOAc in petrol) to yield the title compound (**18**) as an off-white solid (457 mg, 69%). m.p. 106-108 °C (lit: 110-111 °C).

^{1}H NMR (500 MHz, $CDCl_3$) δ 8.35 (d, J = 7.8 Hz, 2H), 8.13 – 8.06 (t, J = 7.8 Hz, 1H), 4.81 (q, J = 8.3 Hz, 4H). **^{13}C NMR** (126 MHz, $CDCl_3$) δ 162.8, 147.4, 138.9, 129.1, 122.9 (q, J = 277.5 Hz), 61.7 (q, J = 37.1 Hz); **IR** ν_{max} (cm^{-1}): 3061, 1757, 1576, 1260, 1153, 962, 760; **HRMS** (ESI) calculated for $C_{11}H_7F_6NNaO_4$ [M+Na]$^+$ 354.0171, found 354.0176.

Diisopropyl pyridine-2,6-dicarboxylate[14] (19)

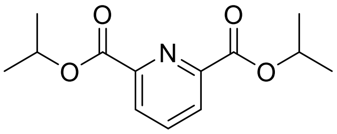

To a solution of pyridine-2,6-dicarbonyl dichloride (408 mg, 2.00 mmol) in CH_2Cl_2 (20 mL) was added isopropanol (459 µL, 6.00 mmol) followed by Et_3N (1.12 mL, 8.00 mmol). The solution was stirred at room temperature for 2 h, diluted with CH_2Cl_2 (50 mL) and washed with water (50 mL). The organic phase was dried over anhydrous $MgSO_4$, filtered and concentrated under reduced pressure. The crude residue was purified by silica gel column chromatography (0-30% EtOAc in petrol) to yield the title compound (**19**) as an off white solid (292 mg, 58%). m.p. 64-65 °C.

^{1}H NMR (400 MHz, $CDCl_3$) δ 8.24 (d, J = 7.8 Hz, 2H), 7.97 (t, J = 7.8 Hz, 1H), 5.33 (hept, J = 6.3 Hz, 2H), 1.43 (d, J = 6.3 Hz, 12H). **^{13}C NMR** (101 MHz, $CDCl_3$) δ 164.2, 149.2, 138.1, 127.7, 70.2, 22.0; **IR** ν_{max} (cm^{-1}): 2926, 1748, 1734, 1717, 1506, 1244, 1105, 754; **HRMS** (ESI) calculated for $C_{13}H_{16}NO_4$ [M+H]$^+$ 252.1230, found 252.1235.

Bis(3-methoxybutyl) pyridine-2,6-dicarboxylate (20)

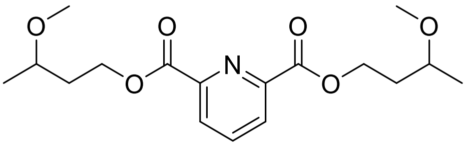

To a solution of pyridine-2,6-dicarbonyl dichloride (500 mg, 2.45 mmol) in CH_2Cl_2 (20 mL) was added 3-methoxy-1-butanol (825 µL, 7.35 mmol) followed by Et_3N (1.37 mL, 9.80 mmol). The solution was stirred at room temperature for 1 h, diluted with CH_2Cl_2 (50 mL) and washed with water (50 mL). The organic phase was dried over anhydrous $MgSO_4$, filtered and concentrated under reduced pressure. The crude residue was purified by silica gel column chromatography (0-30% EtOAc in petrol) to yield the title compound (**20**) as a low melting colourless solid (319 mg, 38%).

^{1}H NMR (400 MHz, $CDCl_3$) δ 8.27 (d, *J* = 7.8 Hz, 2H), 8.00 (dd, *J* = 8.1, 7.5 Hz, 1H), 4.53 (t, *J* = 6.8 Hz, 4H), 3.53 (h, *J* = 6.2 Hz, 2H), 3.35 (s, 6H), 2.06 – 1.92 (m, 4H), 1.23 (d, *J* = 6.1 Hz, 6H); **^{13}C NMR** (101 MHz, $CDCl_3$) δ 164.8, 148.8, 138.3, 127.9, 73.9, 63.4, 56.3, 35.6, 19.3; **IR** ν_{max} (cm^{-1}): 2970, 2928, 1748, 1719, 1325, 1242, 1146, 1082, 752; **HRMS** (ESI) calculated for $C_{17}H_{26}NO_6$ $[M+H]^+$ 340.1755, found 340.1759.

Synthesis of Pyridine-2,6-dicarbaldehyde[17] **(21)**

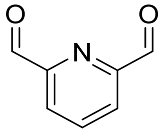

To a solution of 2,6-pyridinedimethanol (417 mg, 3.00 mmol, 1.00 eq) in 1,4-dioxane (10.0 mL) was added SeO_2 (333 mg, 3.00 mmol, 1.00 eq). The resultant mixture was stirred at room temperature for 16 h then concentrated under a stream of nitrogen. The crude product was dissolved in CH_2Cl_2, filtered and concentrated under reduced pressure. Purification by silica gel column chromatography (0-30% EtOAc/Petrol) yielded the title compound (**21**) as a white solid (240 mg, 59%). m.p. 120-122 °C (lit: 122 °C).

^{1}H NMR (500 MHz, $CDCl_3$) δ 10.18 (d, *J* = 0.7 Hz, 2H), 8.19 (dd, *J* = 7.7, 0.7 Hz, 2H), 8.11 – 8.06 (m, 1H). **^{13}C NMR** (126 MHz, $CDCl_3$) δ 192.5, 153.2, 138.5, 125.5; **IR** ν_{max} (cm^{-1}): 2860, 1717, 1348, 1261, 1086, 804; **HRMS** (ESI) calculated for $C_7H_5NNaO_2$ $[M+Na]^+$ 158.0212, found 158.0205.

Supplementary References

1. Klepikova, A. V., Kasianov, A. S., Gerasimov, E. S., Logacheva, M. D. & Penin, A. A. A high resolution map of the Arabidopsis thaliana developmental transcriptome based on RNA-seq profiling. *Plant J.* **88**, 1058–1070 (2016).
2. Zhou, J., Li, B., Qian, Z.-C. & Shi, B.-F. Rhodium(III)-catalyzed oxidative olefination of picolinamides: Convenient synthesis of 3-alkenylpicolinamides. *Adv. Synth. Catal.* **356**, 1038–1046 (2014).
3. Le Borgne, T. *et al.* Monometallic lanthanide complexes with tridentate 2,6-dicarboxamidopyridine ligands. Influence of peripheral substitutions on steric congestion and antenna effect. *J. Chem. Soc. Dalt. Trans.* 3856–3868 (2003) doi:10.1039/B307413G.
4. Buhleier, E., Wehner, W. & Vögtle, F. Zur gehinderten Rotation an der Carbonamid-Bindung von Kronenether-, Cryptand- und verwandten Systemen - Elektronische und sterische Substituenteneinflusse. *Chem. Ber.* **112**, 559–566 (1979).
5. Okamoto, I. *et al.* Acid-induced conformational alteration of cis-preferential aromatic amides bearing N-methyl-N-(2-pyridyl) moiety. *Tetrahedron* **67**, 8536–8543 (2011).
6. Speelman, J. C. & Kellogg, R. M. Behavior of Pyridinium Salts Obtained from Derivatives of Pyridinedicarboxylic Acids in Basic Solutions. Addition of Hydroxide or Alkoxide To Form 1.2-Dihvdronvridine Intermediates. *J. Org. Chem.* **55**, 647–653 (1990).
7. Ivchenko, P. V., Nifant'Ev, I. E. & Buslov, I. V. A convenient approach for the synthesis of 2,6-diformyl- and 2,6-diacetylpyridines. *Tetrahedron Lett.* **54**, 217–219 (2013).
8. Chauhan, A. *et al.* A small molecule peptidomimetic that binds to c-KIT1 G-quadruplex and exhibits antiproliferative properties in cancer cells. *Bioorganic Med. Chem.* **22**, 4422–4429 (2014).
9. Lewandowski, B. & Jarosz, S. Amino-acid templated assembly of sucrose-derived macrocycles. *Org. Lett.* **12**, 2532–2535 (2010).
10. Bremer, A. *et al.* 2,6-Bis(5-(2,2-dimethylpropyl)-1H-pyrazol-3-yl)pyridine as a ligand for efficient actinide(III)/lanthanide(III) separation. *Inorg. Chem.* **51**, 5199–5207 (2012).
11. Zhu, H. *et al.* Synthesis, crystal structure, and different local conformations of pyridine-imide oligomers. *Tetrahedron* **67**, 8458–8464 (2011).
12. Iwasaki, T., Maegawa, Y., Hayashi, Y., Ohshima, T. & Mashima, K. Transesterification of various methyl esters under mild conditions catalyzed by tetranuclear zinc cluster. *J. Org. Chem.* **73**, 5147–5150 (2008).
13. Massah, A. R., Kalbasi, R. J., Khalifesoltani, M. & Moshtagh Kordesofla, F. ZSM-5-SO 3 H: An Efficient Catalyst for Acylation of Sulfonamides Amines, Alcohols, and Phenols under Solvent-Free Conditions . *ISRN Org. Chem.* **2013**, 1–12 (2013).
14. Steinkamp, T. & Karst, U. Detection scheme for bioassays based on 2,6-pyridinedicarboxylic acid derivatives and enzyme-amplified lanthanide luminescence. *Anal. Chim. Acta* **526**, 27–34 (2004).
15. Rujhofer, W., Miiller, W. M. & Vögtle, F. Nichtcyclische Kronenether-artige Ester und ihre. *Chem. Ber.* **112**, 2095–2119 (1979).
16. Singh, G. & Rani, S. An expedient 'click' approach for the synthetic evaluation of ester-triazole-tethered organosilica conjugates. *Appl. Organomet. Chem.* **32**,

e4028 (2018).
17. Salanouve, E. *et al.* Tandem C-H activation/arylation catalyzed by low-valent iron complexes with bisiminopyridine ligands. *Chem. Eur. J.* **20**, 4754–4761 (2014).

Supplementary Figure 7. NMR spectra of compounds.

Compound 1

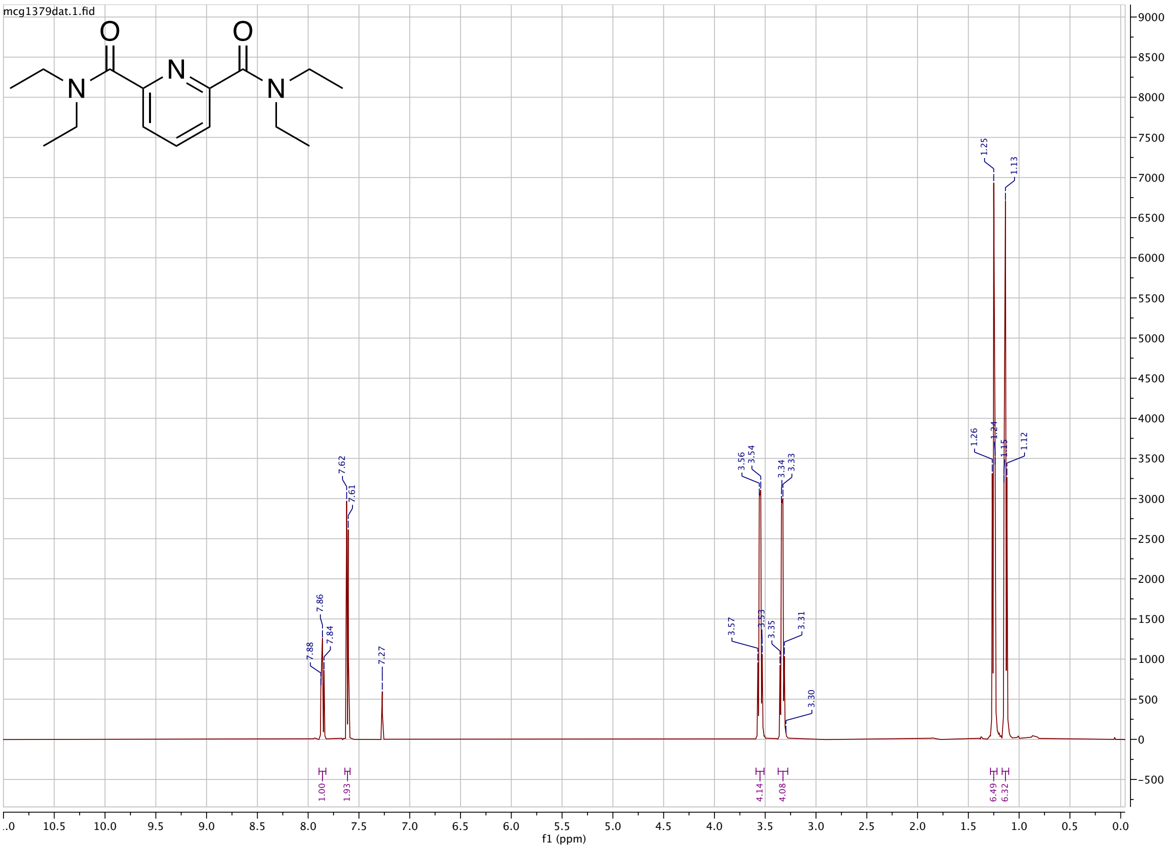

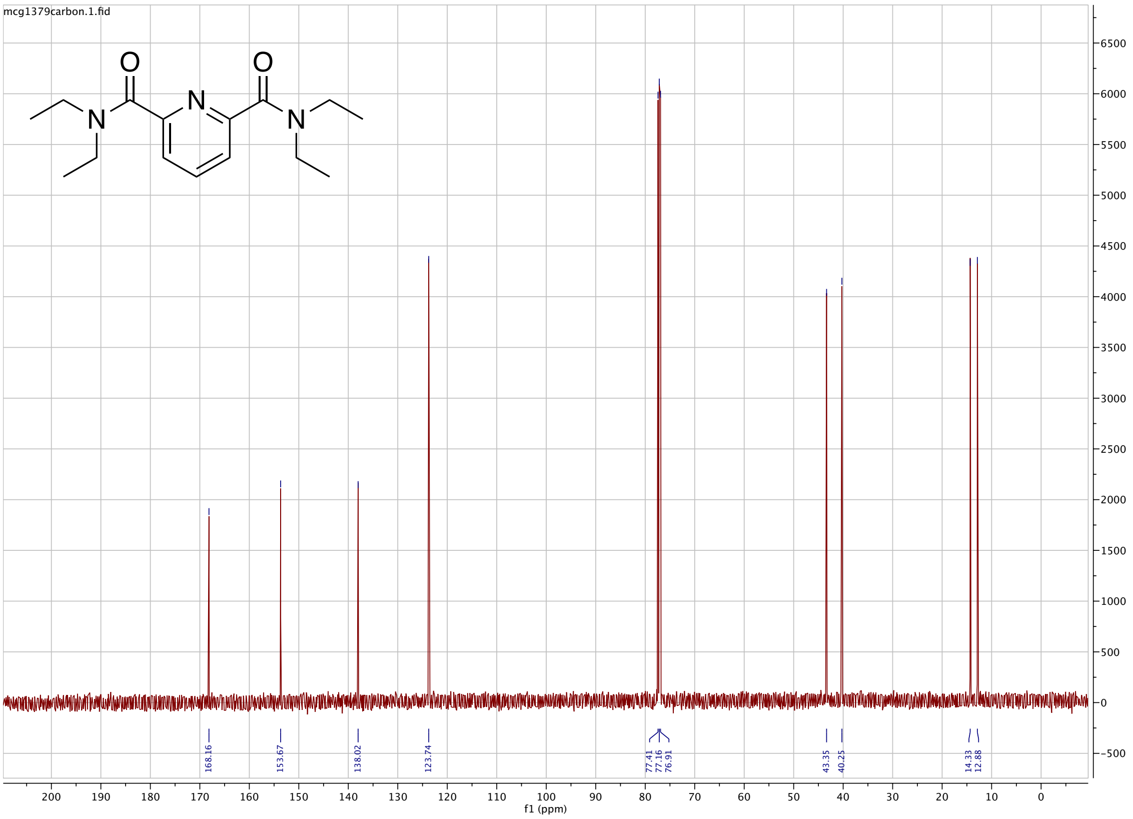

Compound 2

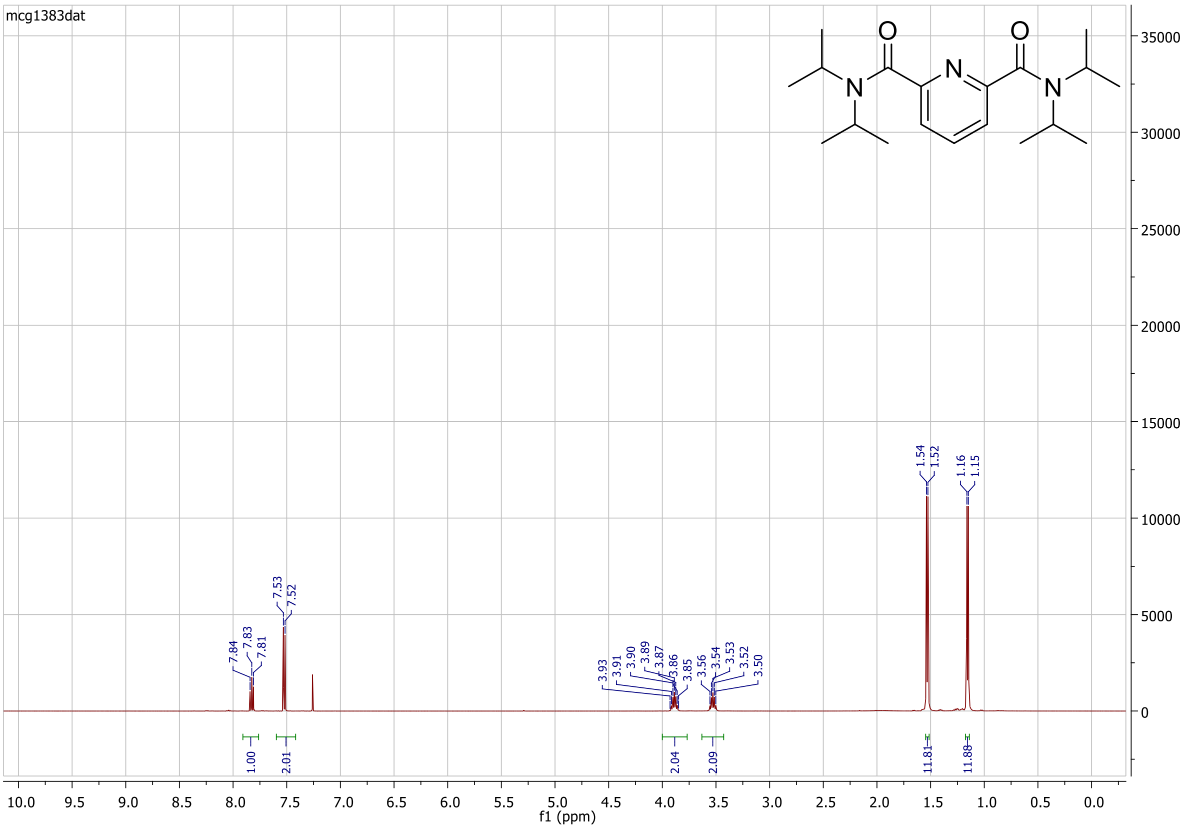
mcg1383dat
7.84
7.83
7.81
7.53
7.52
3.93
3.91
3.90
3.89
3.87
3.86
3.85
3.56
3.54
3.53
3.52
3.50
1.54
1.52
1.16
1.15
1.00
2.01
2.04
2.09
11.81
11.88
f1 (ppm)
O
N
O
N
N

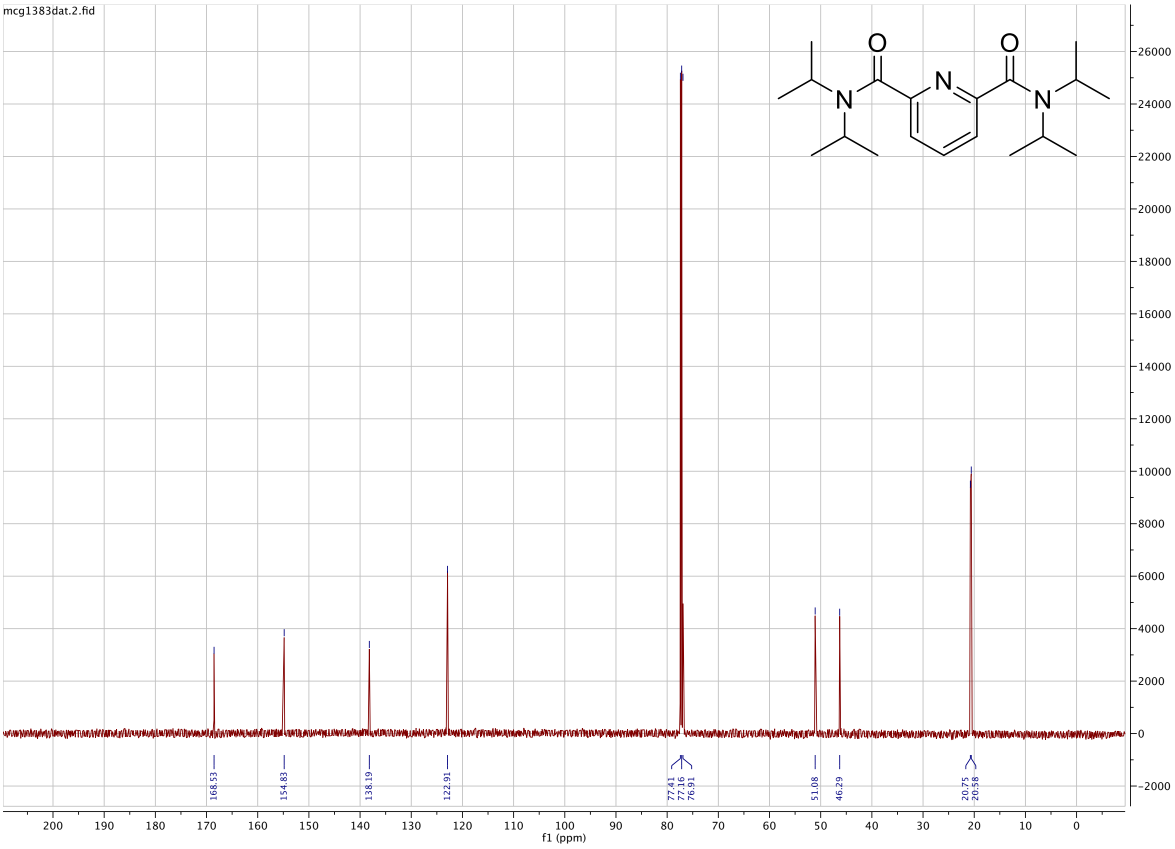
mcg1383dat.2.fid
168.53
154.83
138.19
122.91
77.41
77.16
76.91
51.08
46.29
20.75
20.58
f1 (ppm)
O
N
O
N
N

Compound 3

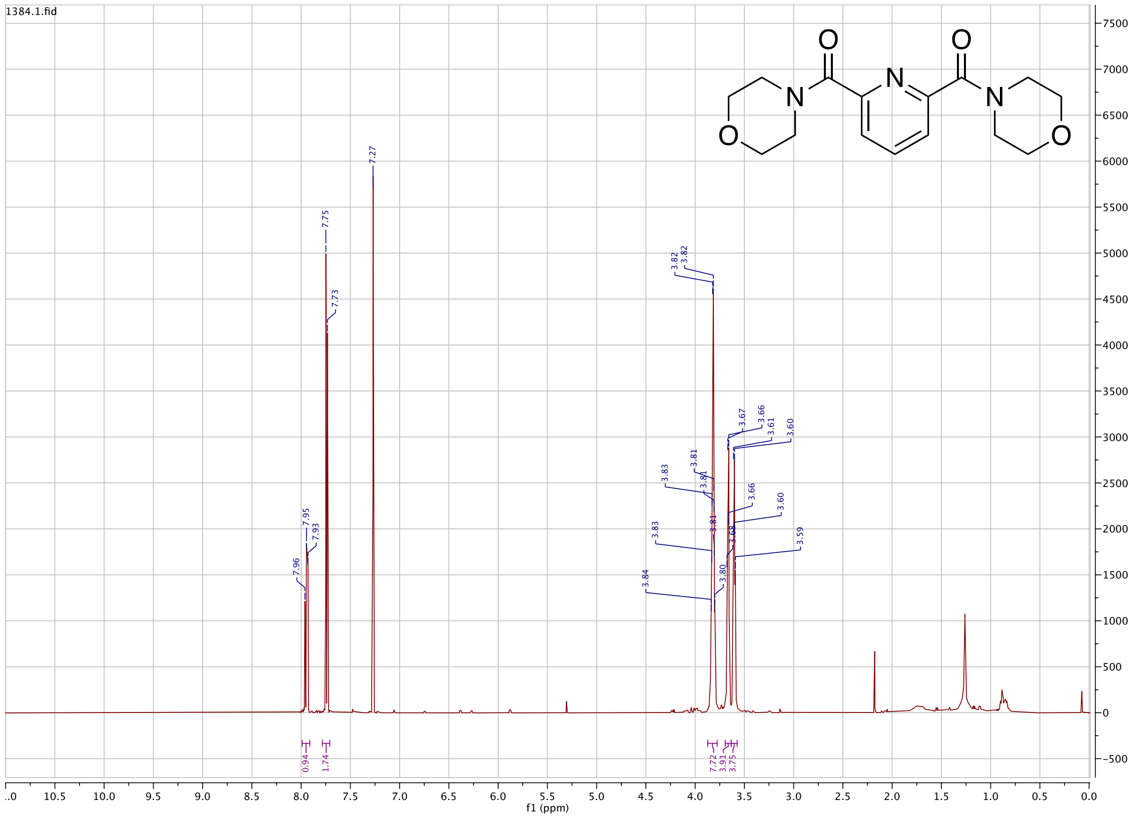

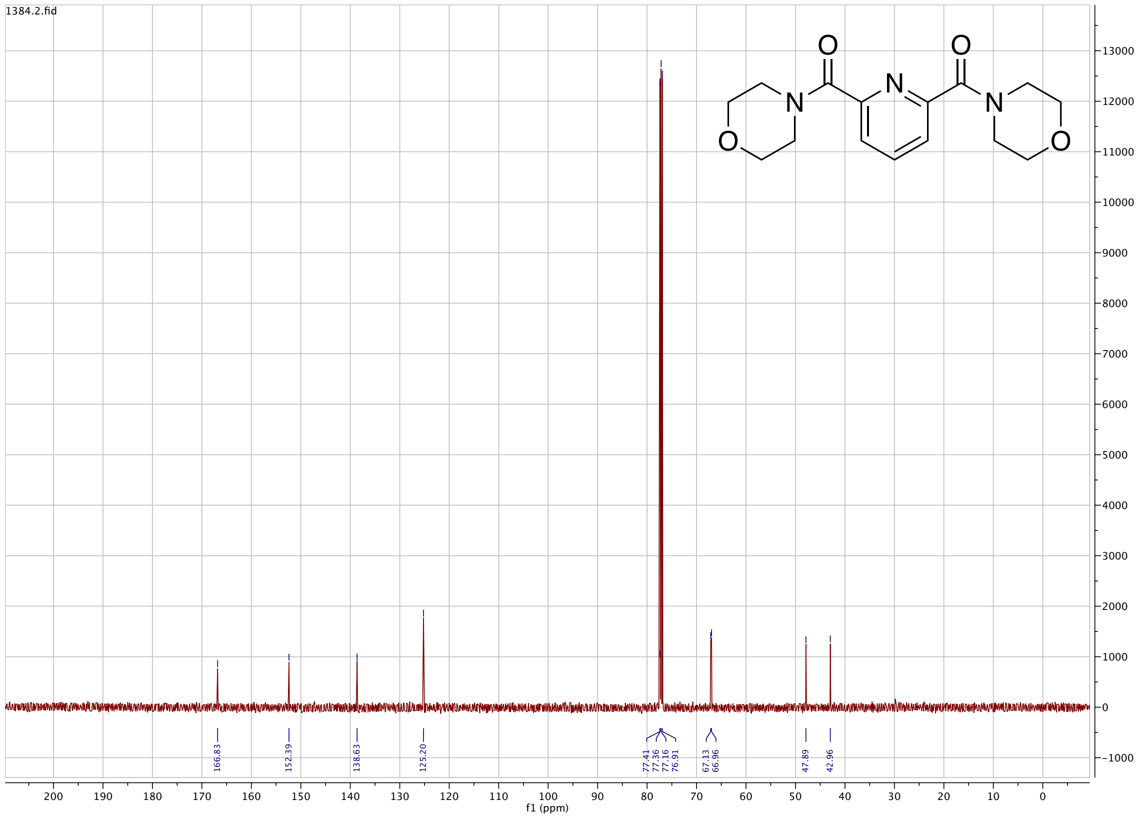

Compound 4

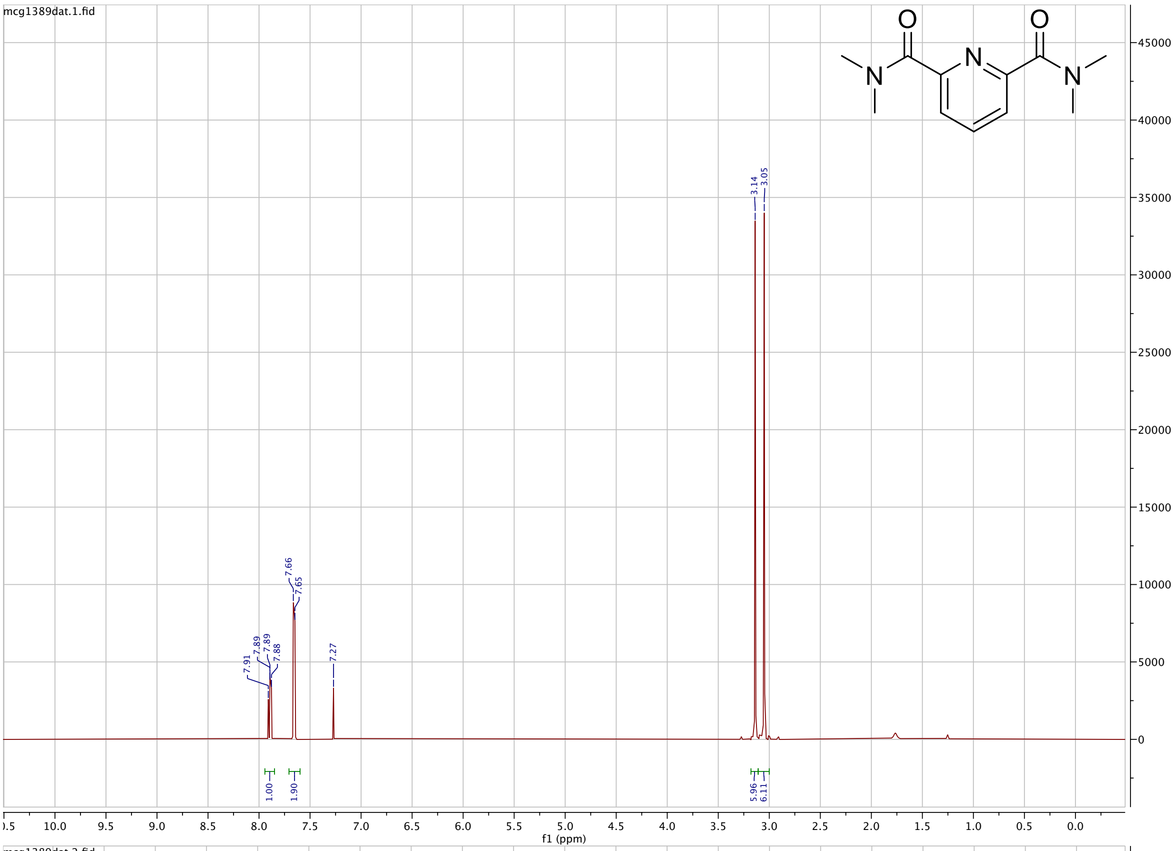
mcg1389dat.1.fid
7.91
7.89
7.89
7.88
7.66
7.65
7.27
3.14
3.05
1.00
1.90
5.96
6.11
f1 (ppm)

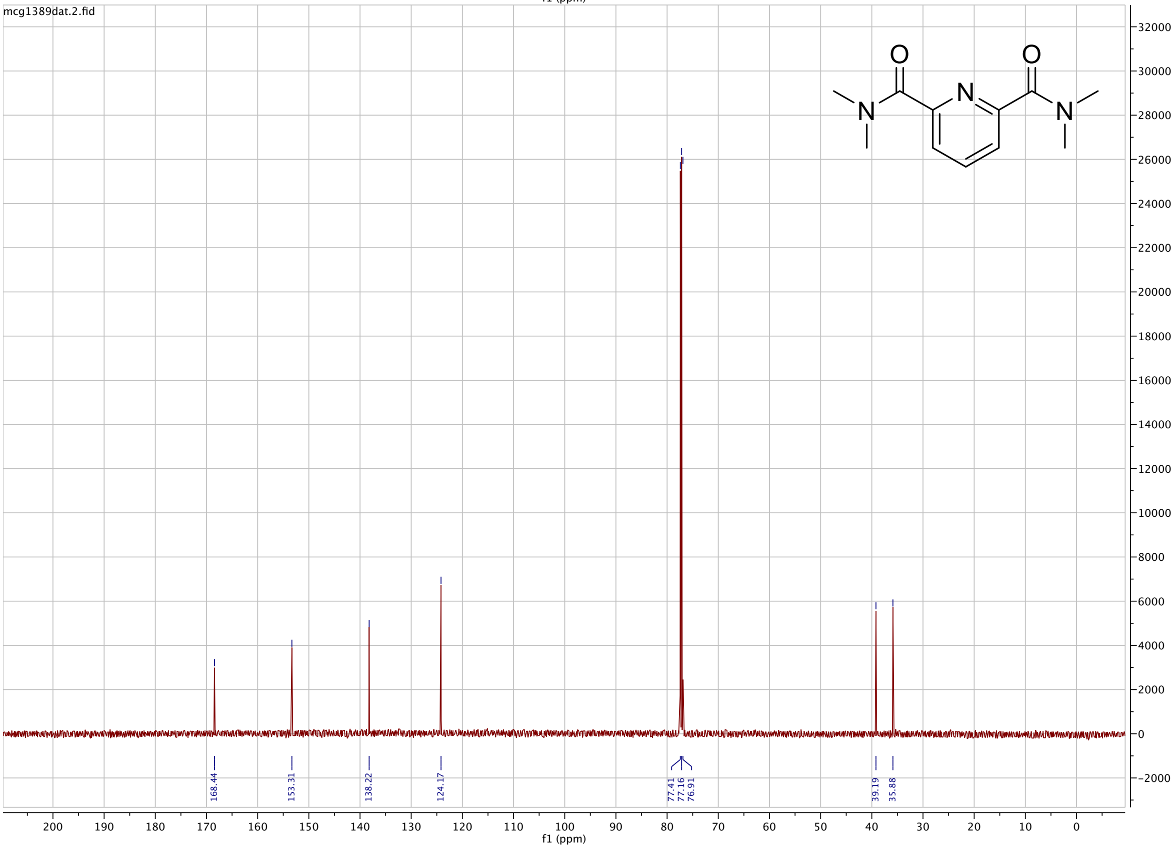
mcg1389dat.2.fid
168.44
153.31
138.22
124.17
77.41
77.16
76.91
39.19
35.88
f1 (ppm)

Compound 5

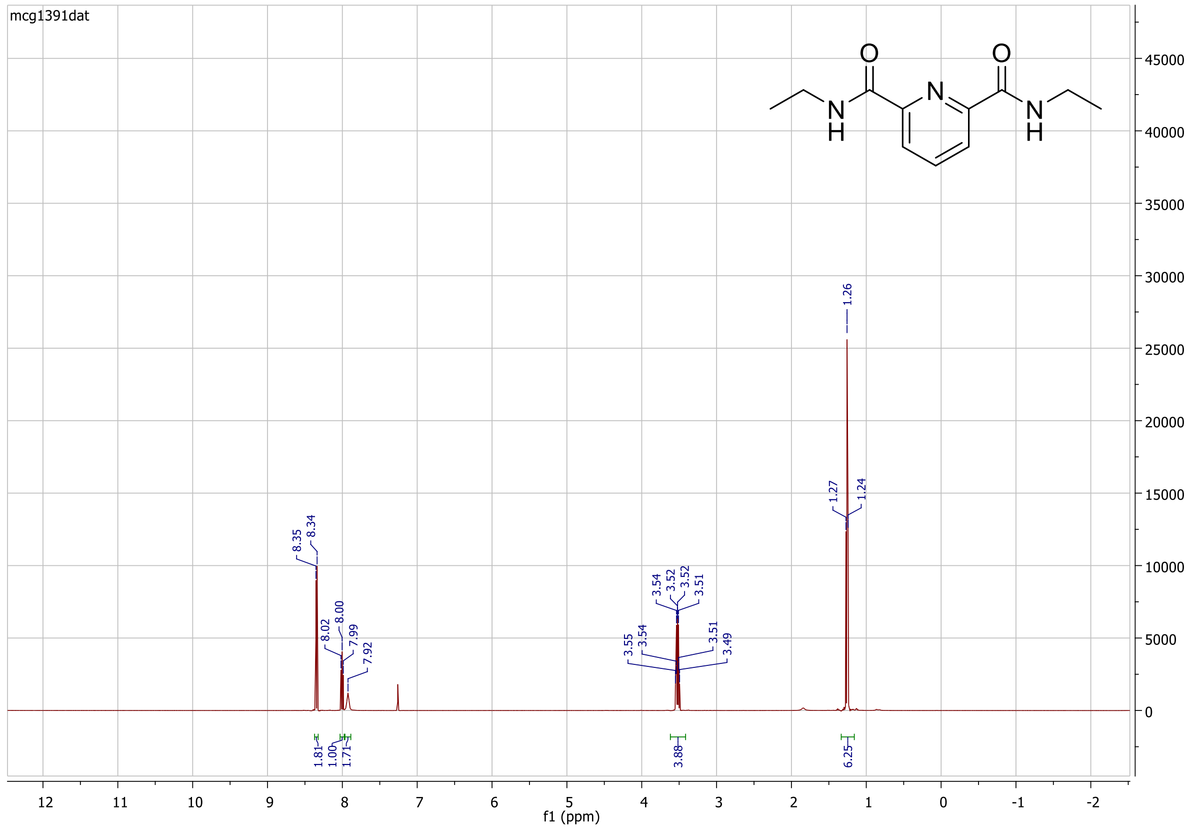
mcg1391dat
8.35
8.34
8.02
8.00
7.99
7.92
3.55
3.54
3.54
3.52
3.52
3.51
3.51
3.49
1.27
1.26
1.24
1.81
1.00
1.71
3.88
6.25
f1 (ppm)

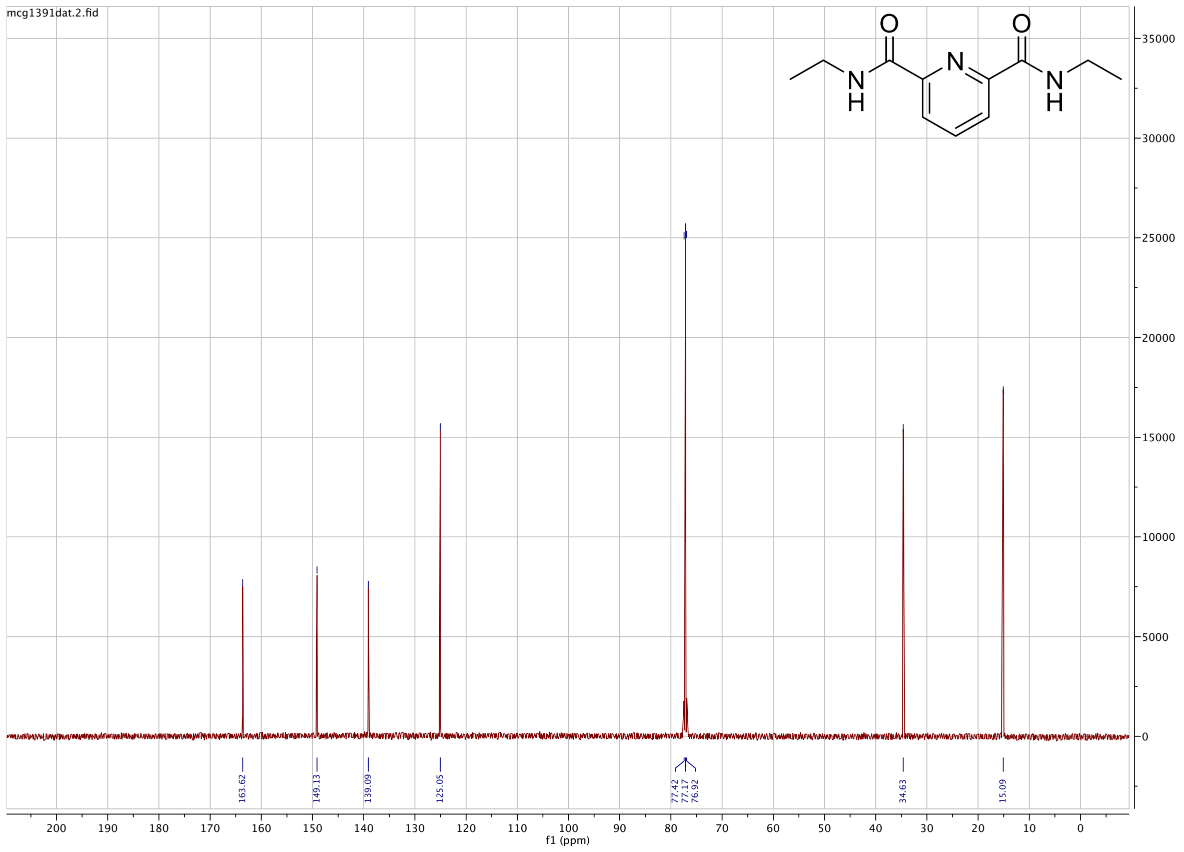
mcg1391dat.2.fid
163.62
149.13
139.09
125.05
77.42
77.17
76.92
34.63
15.09
f1 (ppm)

Compound 6

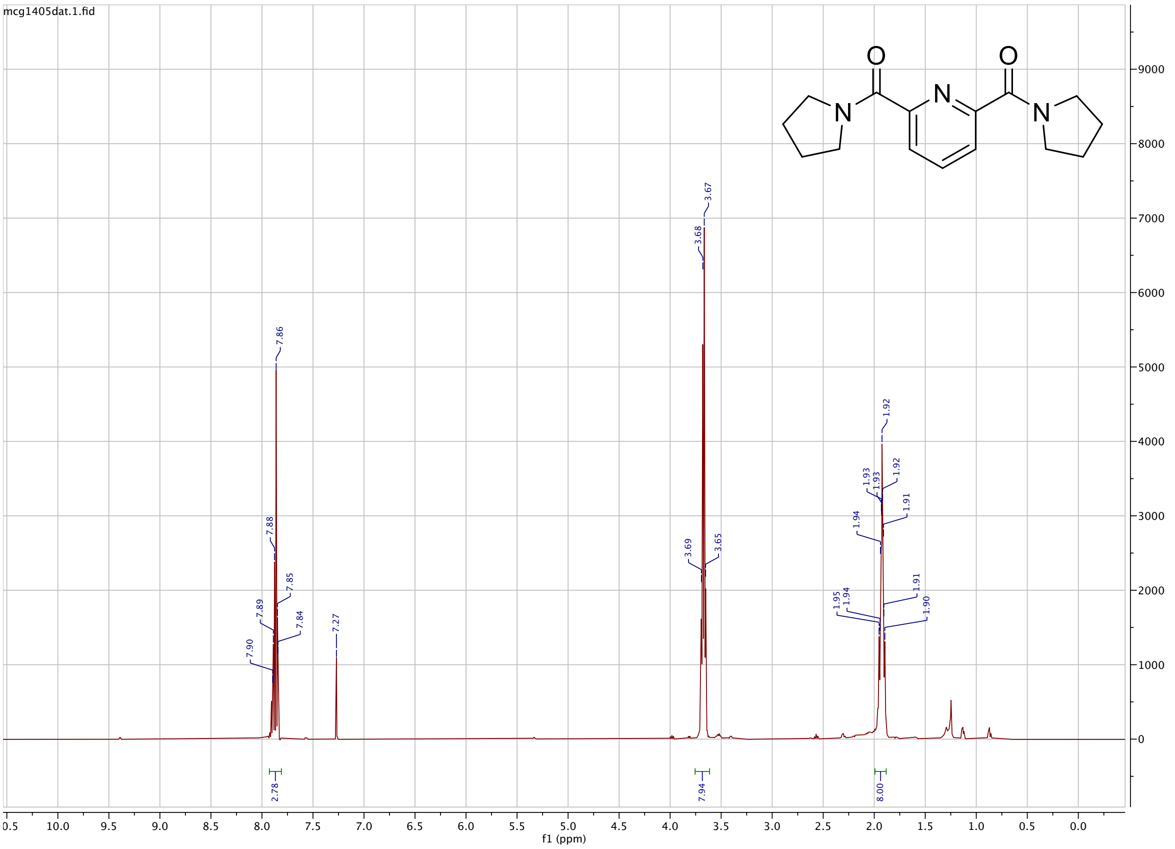
mcg1405dat.1.fid
7.90
7.89
7.88
7.86
7.85
7.84
7.27
3.69
3.68
3.67
3.65
1.95
1.94
1.94
1.93
1.93
1.92
1.92
1.91
1.91
1.90
2.78
7.94
8.00
f1 (ppm)

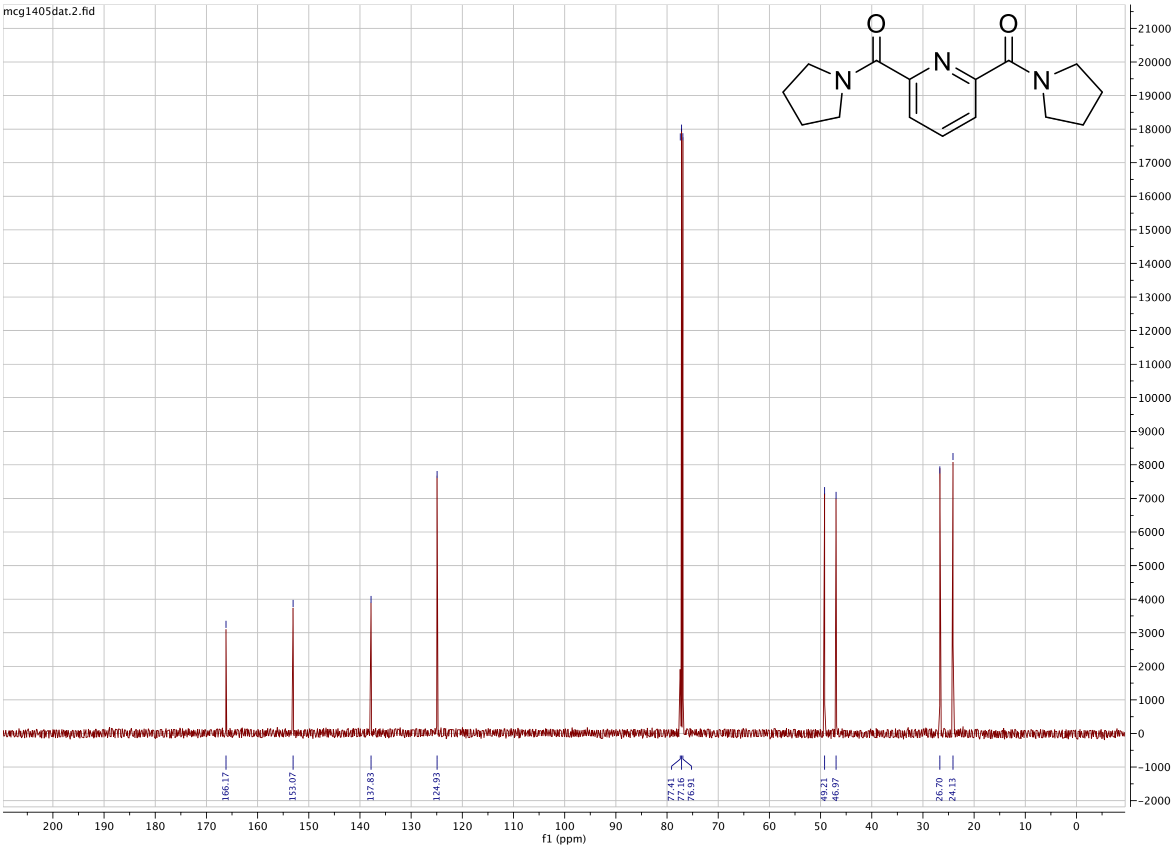
mcg1405dat.2.fid
166.17
153.07
137.83
124.93
77.41
77.16
76.91
49.21
46.97
26.70
24.13
f1 (ppm)

Compound 7

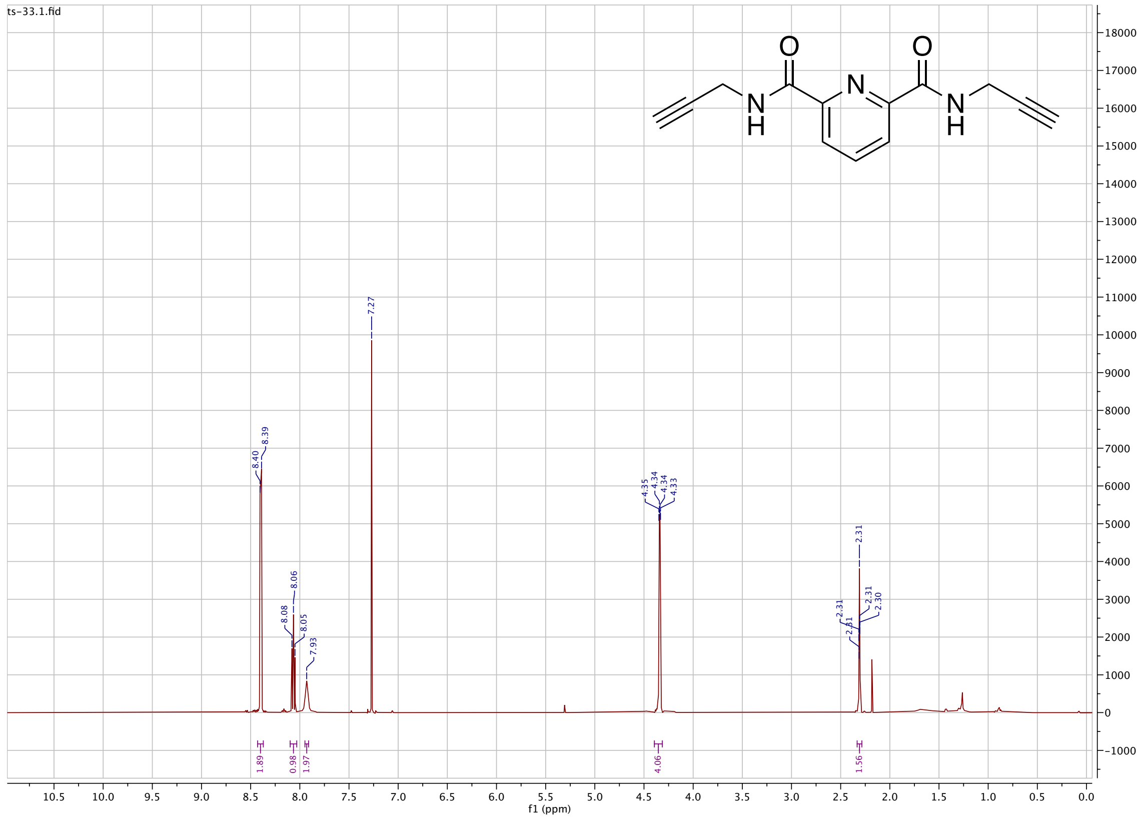

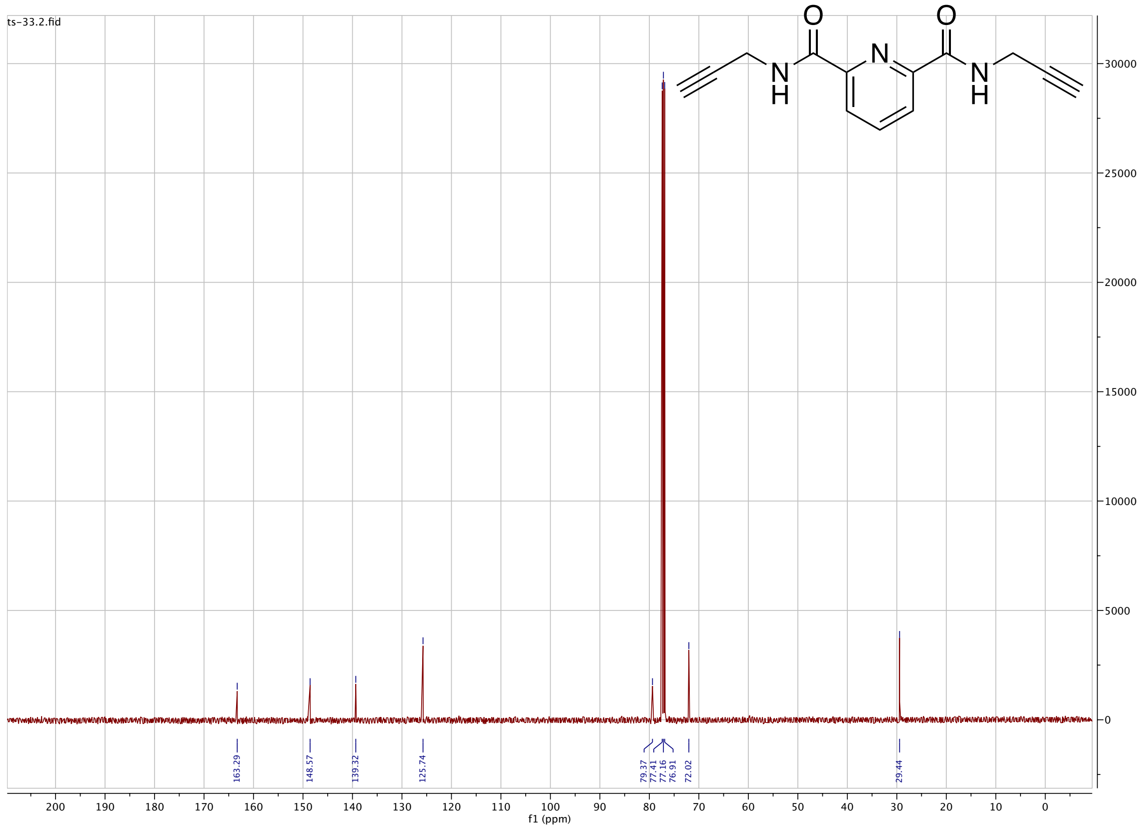

Compound 8

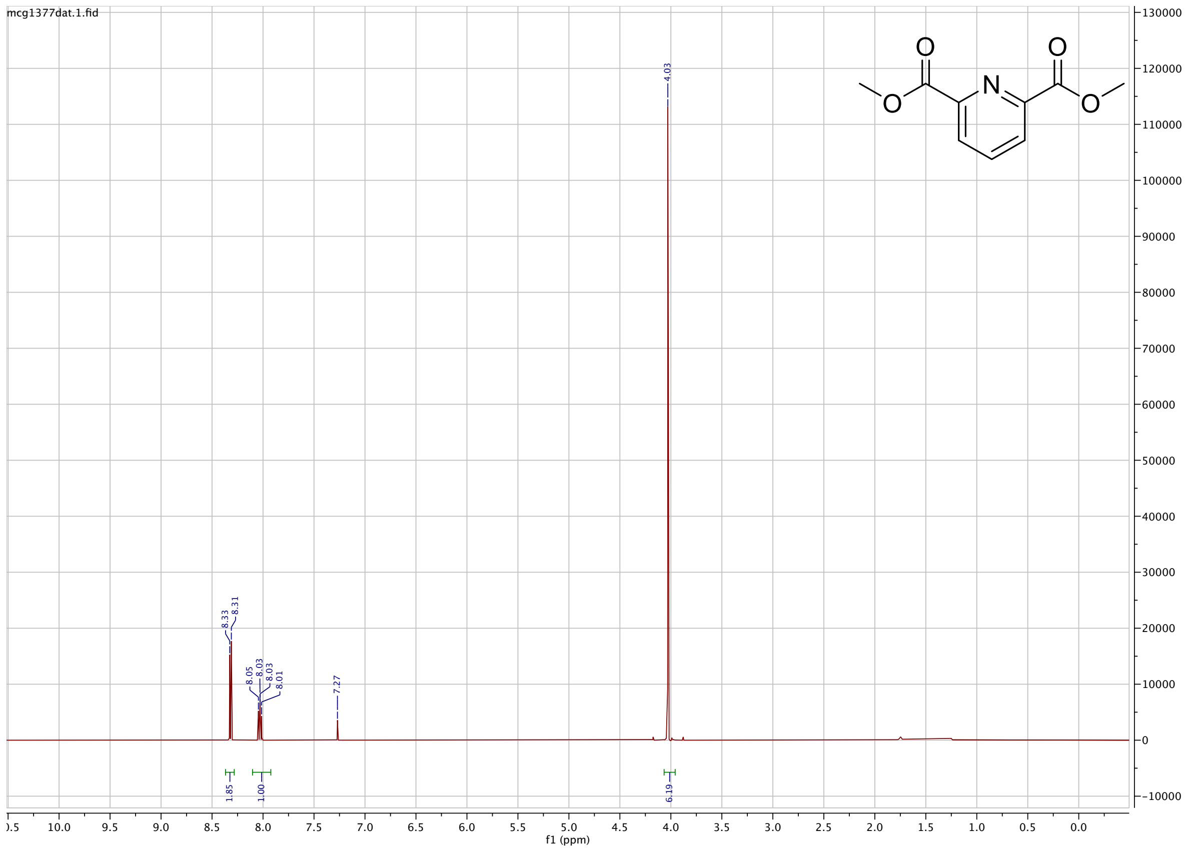
mcg1377dat.1.fid
8.33
8.31
8.05
8.03
8.03
8.01
7.27
4.03
1.85
1.00
6.19
f1 (ppm)

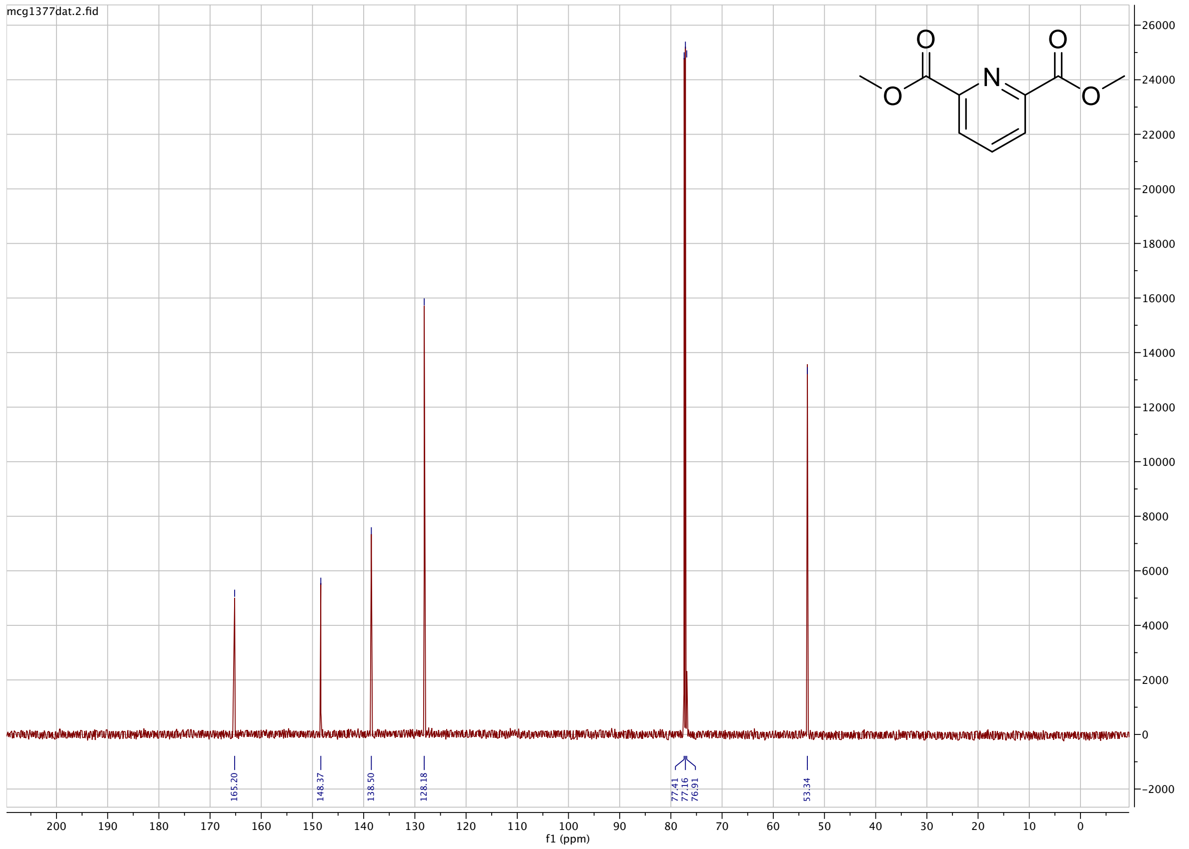
mcg1377dat.2.fid
165.20
148.37
138.50
128.18
77.41
77.16
76.91
53.34
f1 (ppm)

Compound 9

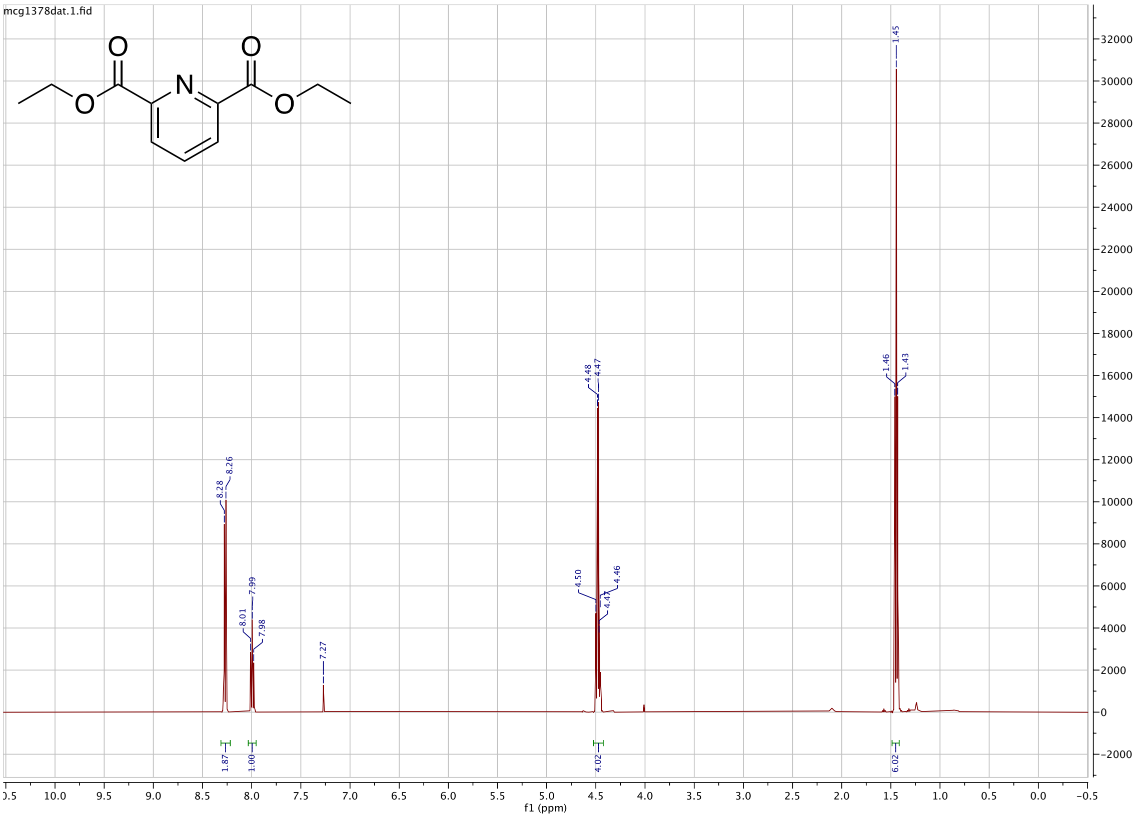
mcg1378dat.1.fid
N
O
O
O
O
8.28
8.26
8.01
7.99
7.98
7.27
4.50
4.48
4.47
4.47
4.46
1.46
1.45
1.43
1.87
1.00
4.02
6.02
f1 (ppm)

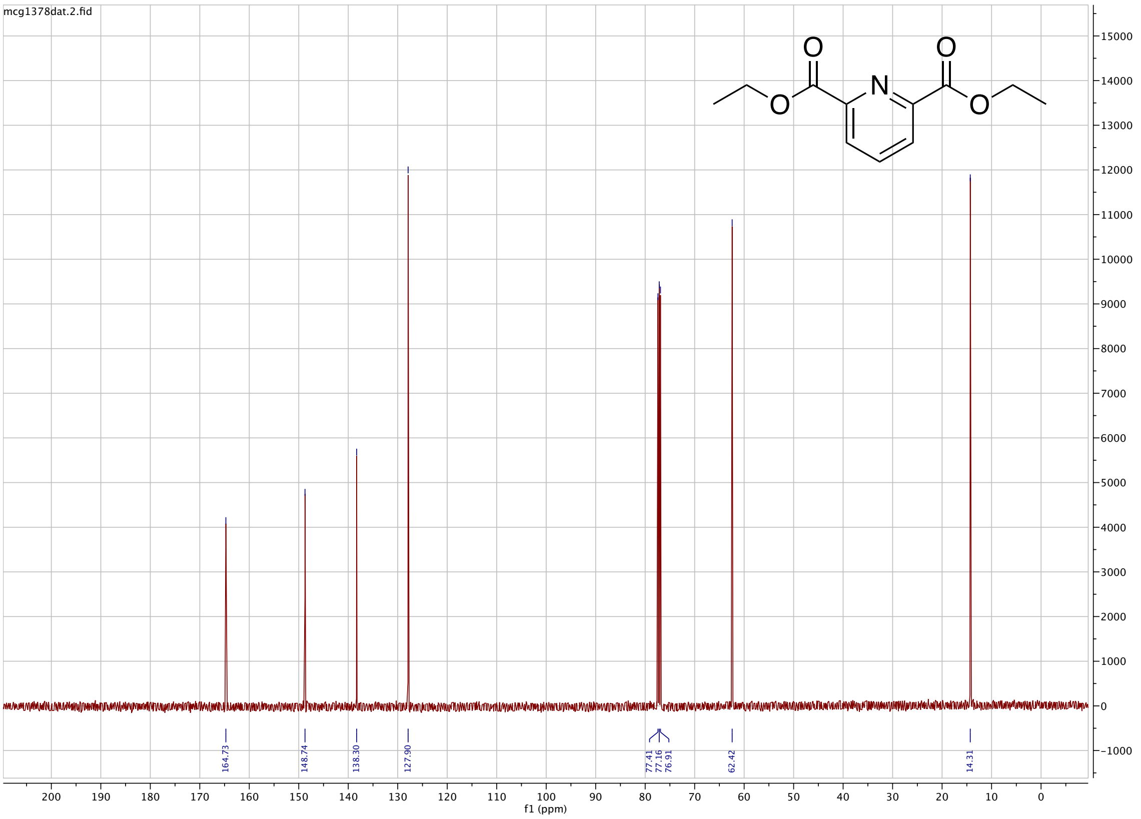
mcg1378dat.2.fid
N
O
O
O
O
164.73
148.74
138.30
127.90
77.41
77.16
76.91
62.42
14.31
f1 (ppm)

Compound 10

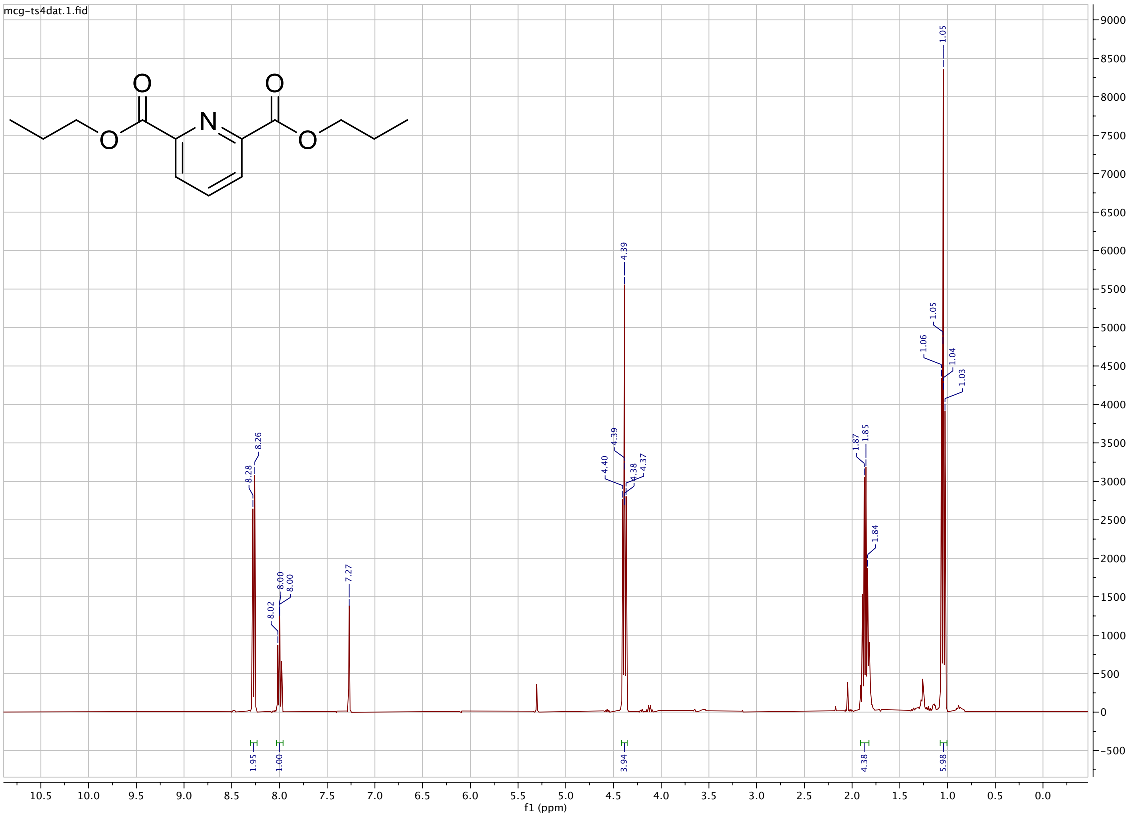

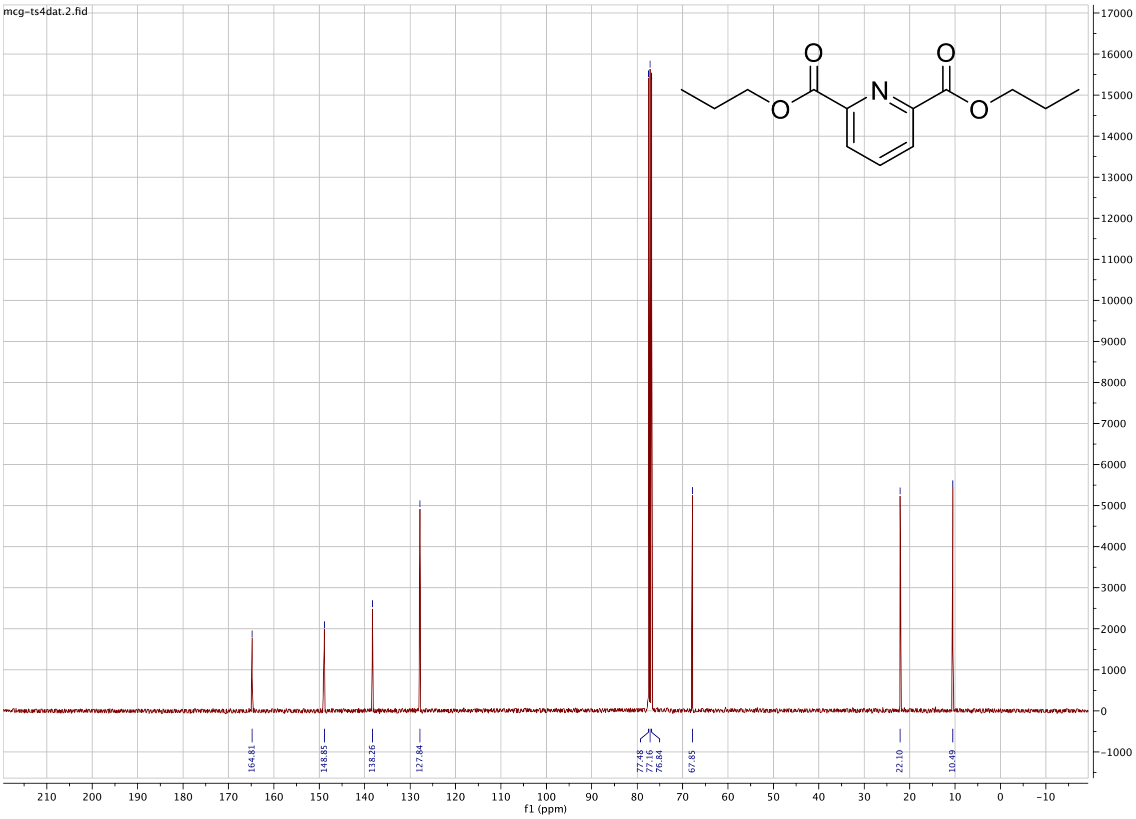

Compound 11

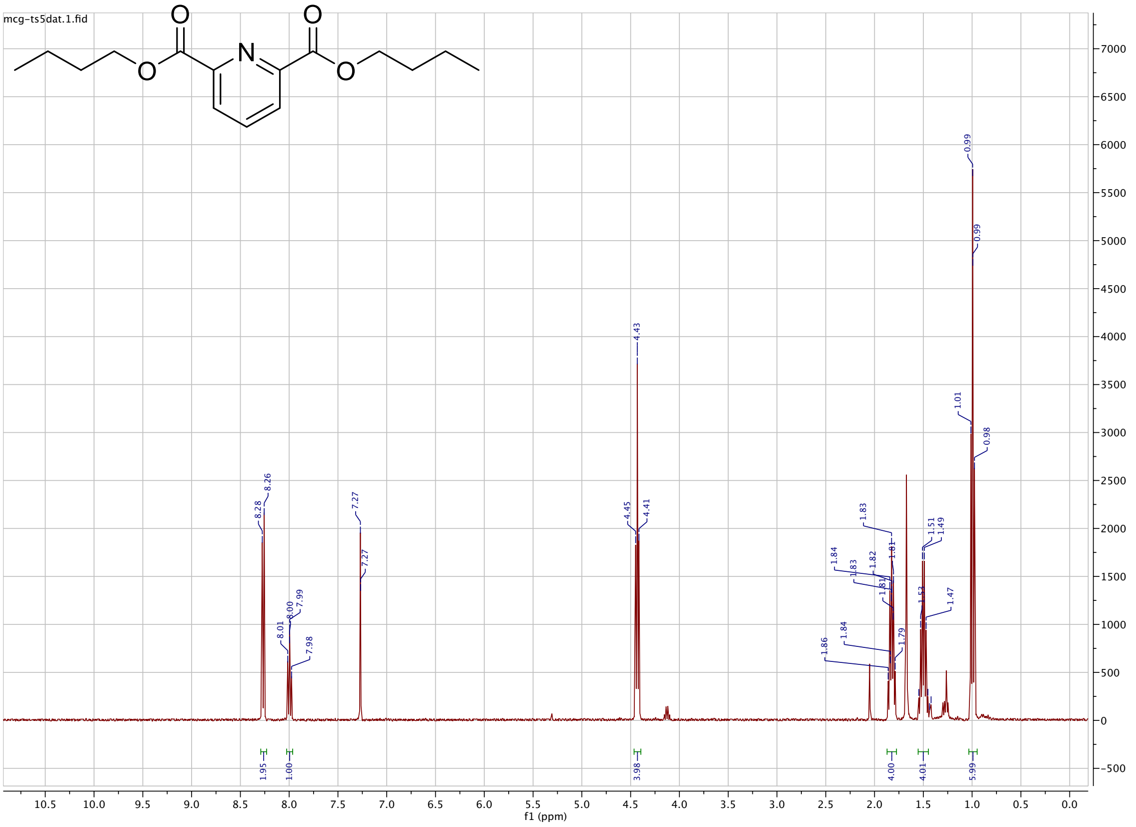

mcg-ts5dat.1.fid
f1 (ppm)

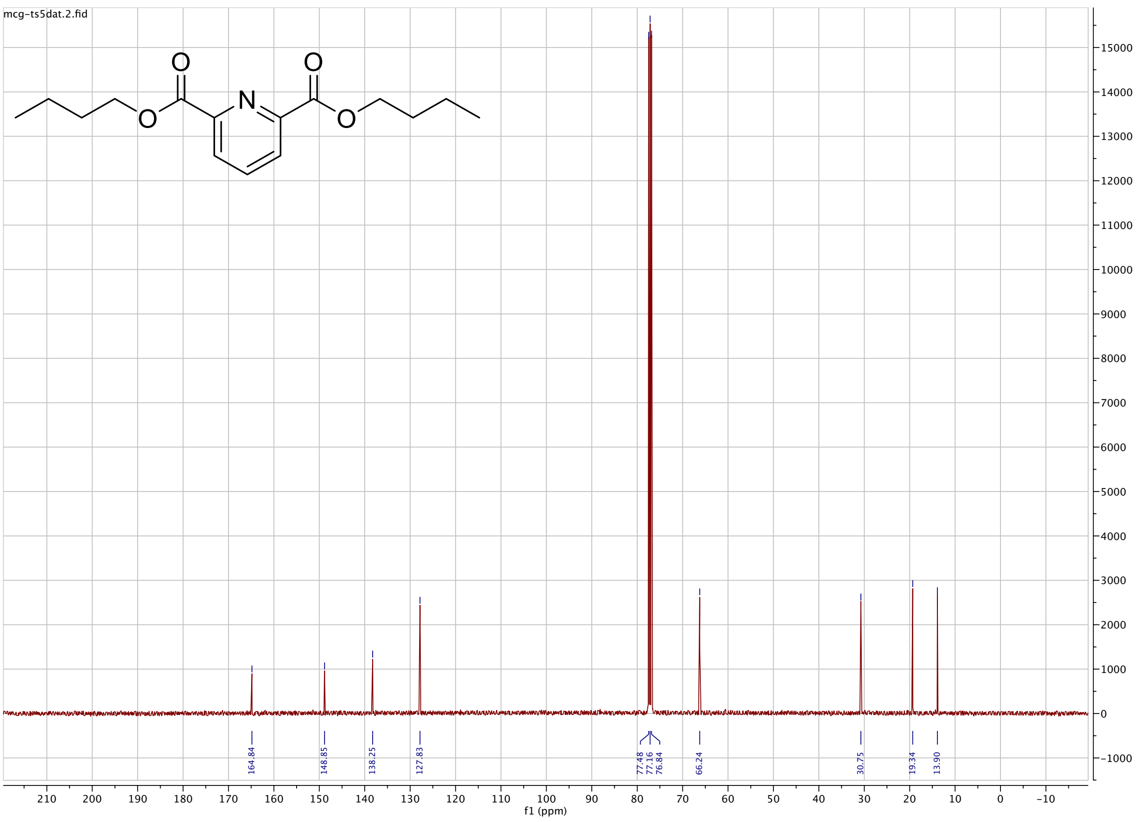

mcg-ts5dat.2.fid
164.84
148.85
138.25
127.83
77.48
77.16
76.84
66.24
30.75
19.34
13.90
f1 (ppm)

Compound 12

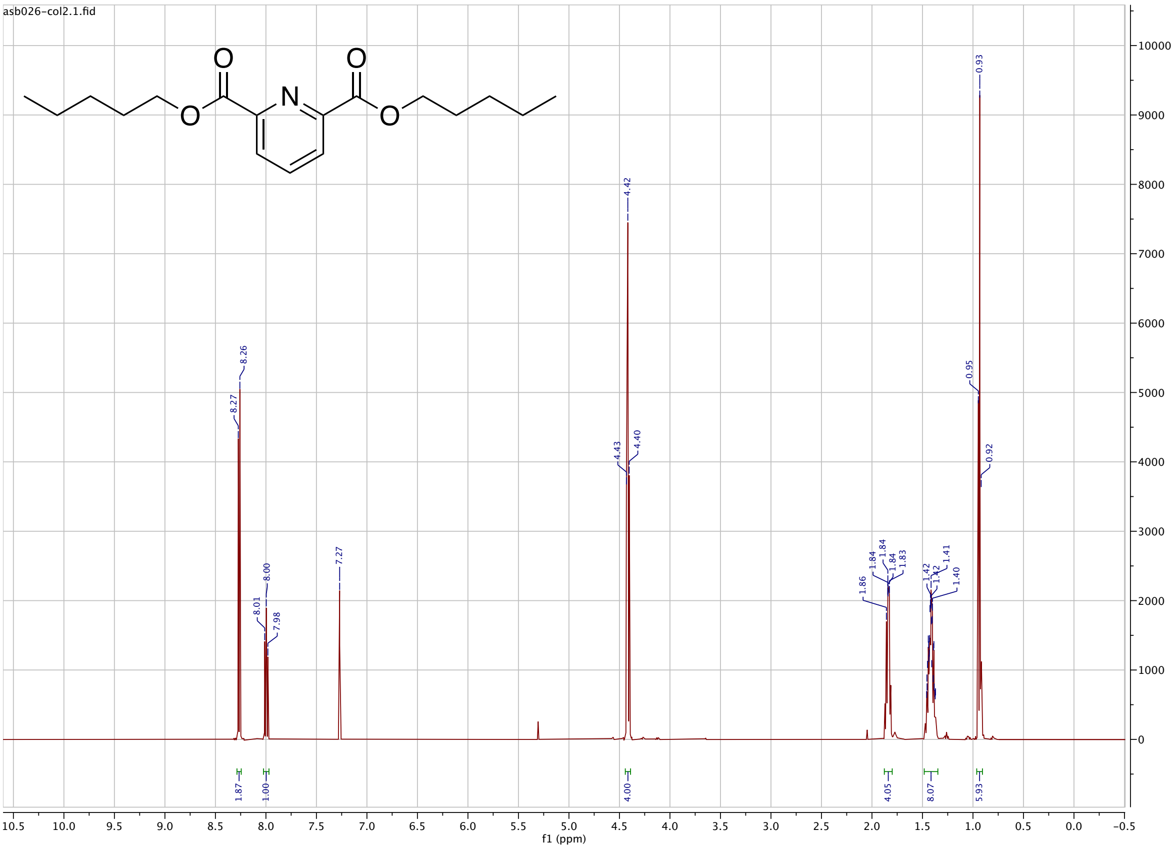

asb026-col2.1.fid
N
O
O
O
O
8.27
8.26
8.01
8.00
7.98
7.27
4.43
4.42
4.40
1.86
1.84
1.84
1.84
1.83
1.42
1.42
1.41
1.40
0.95
0.93
0.92
1.87
1.00
4.00
4.05
8.07
5.93
f1 (ppm)

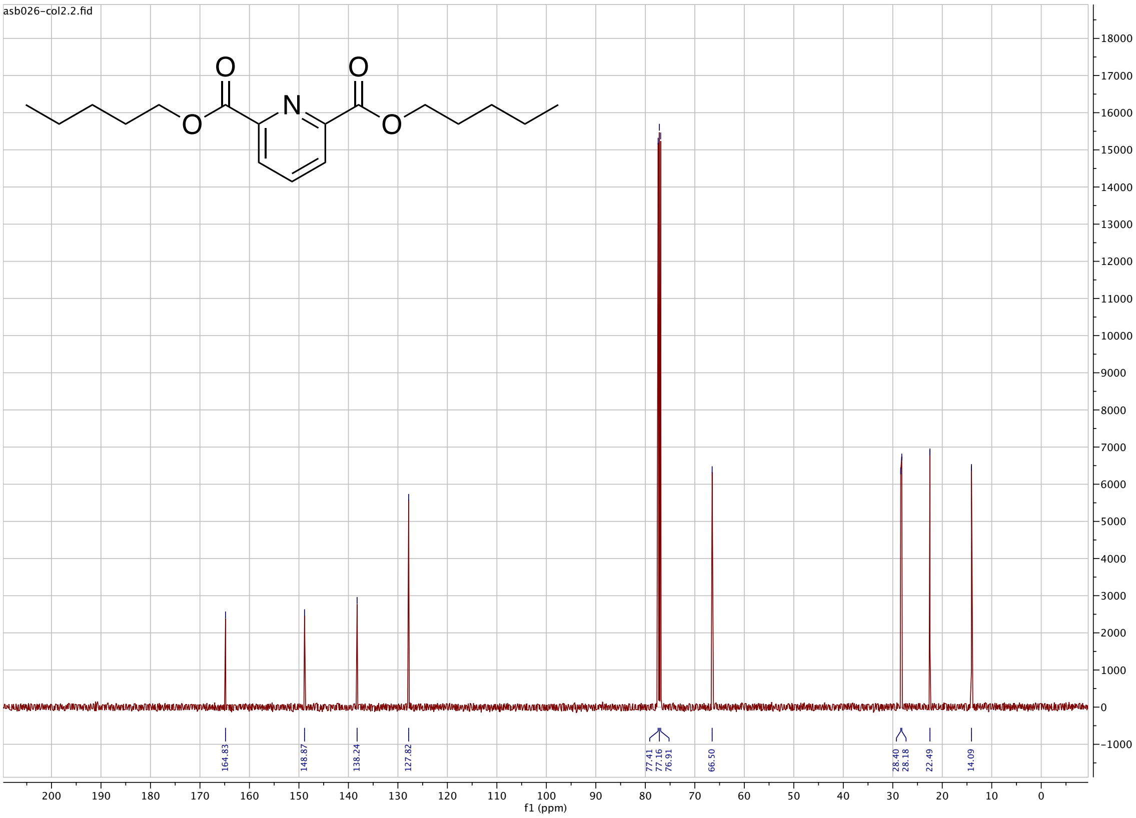

asb026-col2.2.fid
N
O
O
O
O
164.83
148.87
138.24
127.82
77.41
77.16
76.91
66.50
28.40
28.18
22.49
14.09
f1 (ppm)

Compound 13

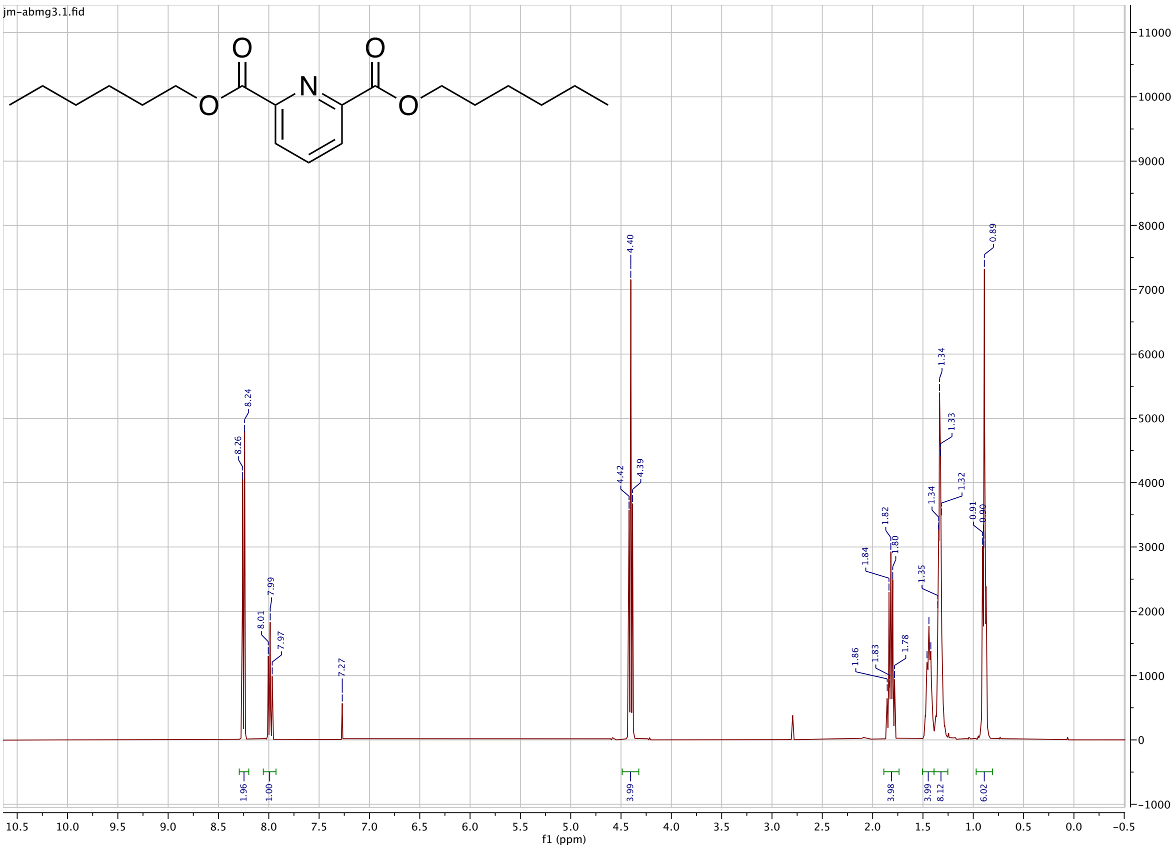

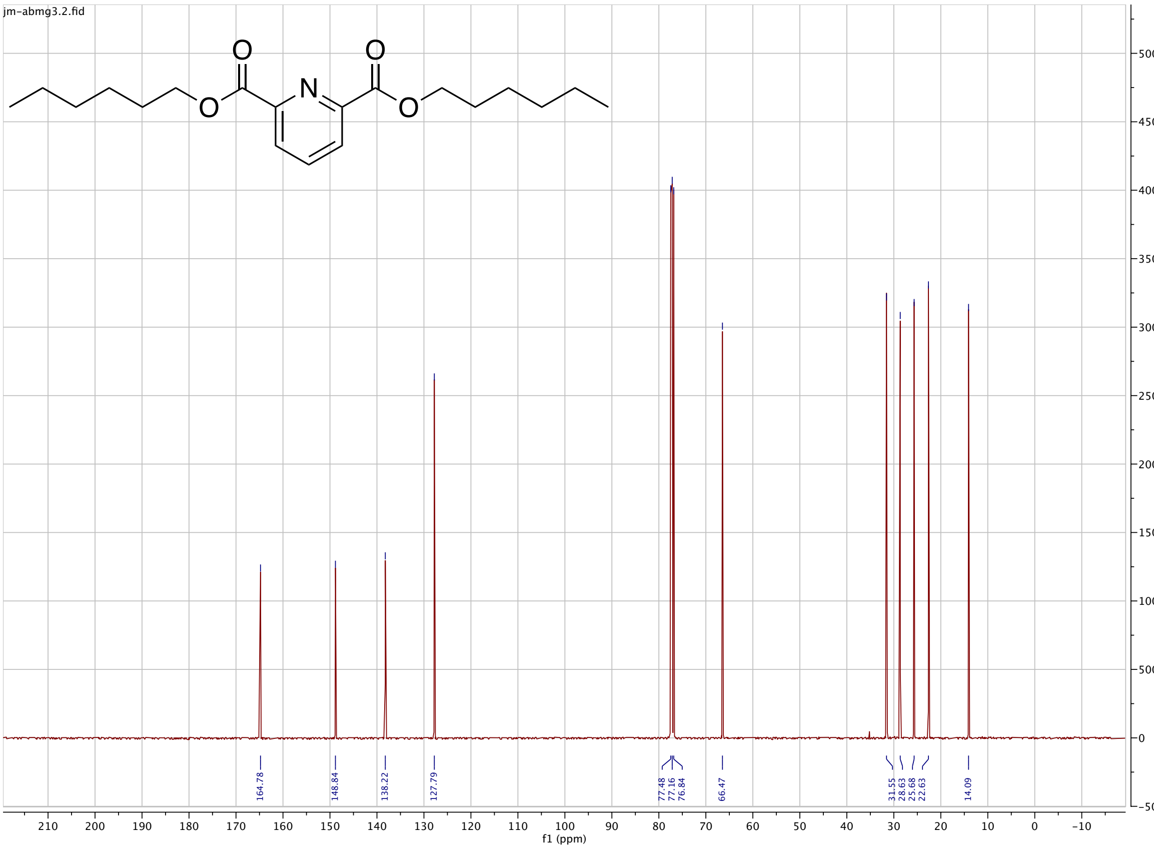

Compound 14

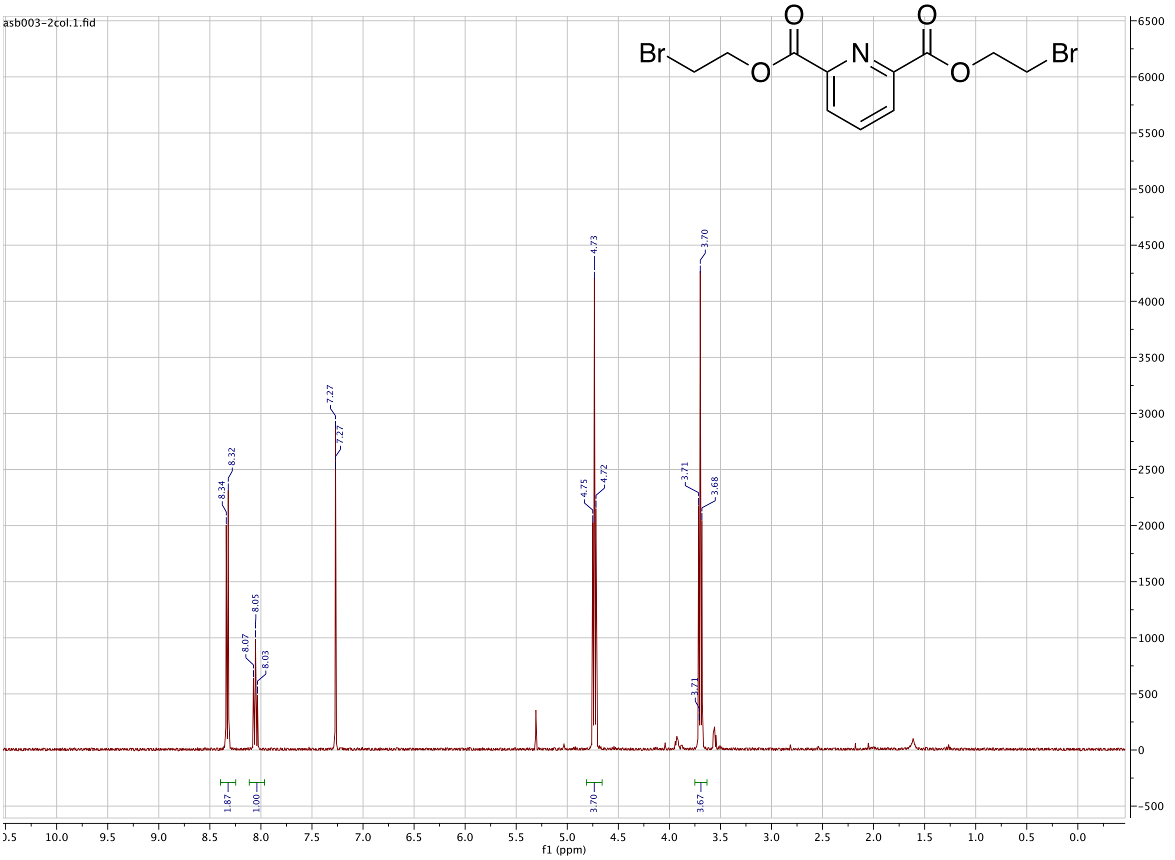
asb003-2col.1.fid
Br
O
O
N
O
O
Br
8.34
8.32
8.07
8.05
8.03
7.27
7.27
4.75
4.73
4.72
3.71
3.70
3.68
3.71
1.87
1.00
3.70
3.67
f1 (ppm)

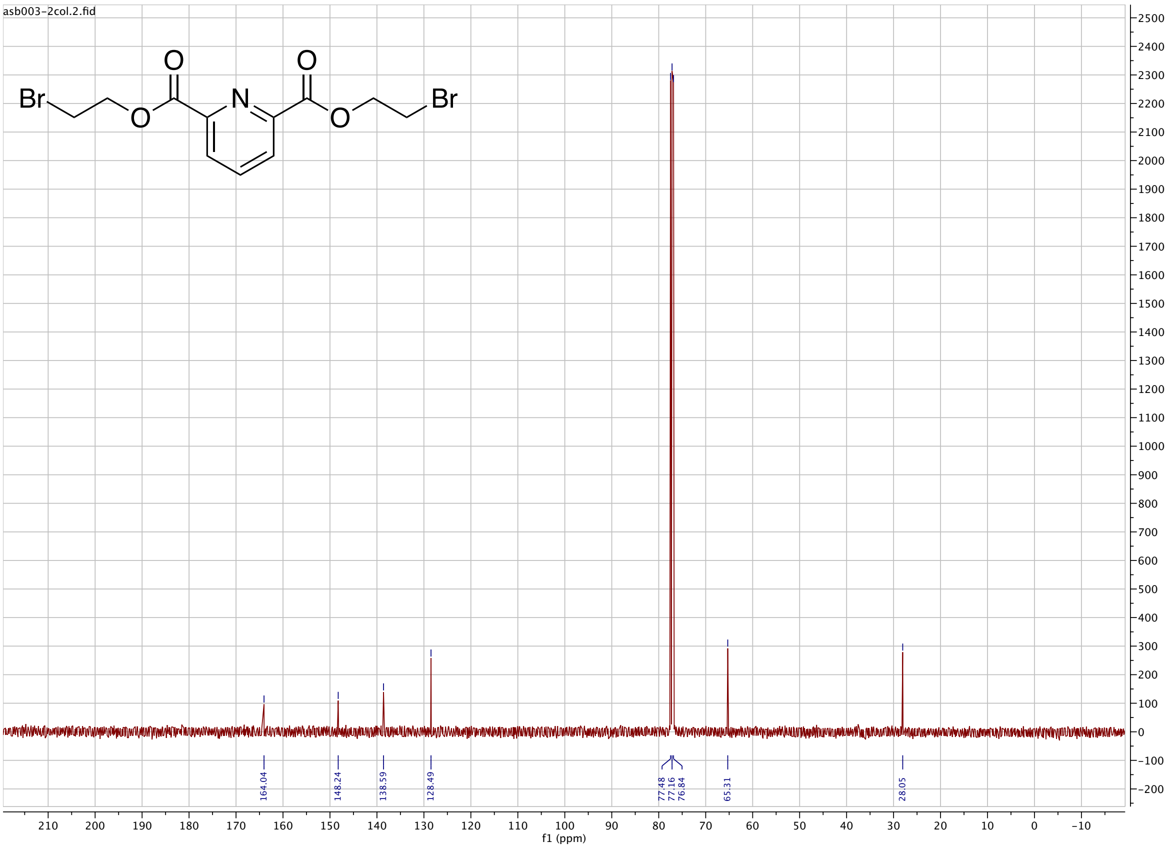
asb003-2col.2.fid
Br
O
O
N
O
O
Br
164.04
148.24
138.59
128.49
77.48
77.16
76.84
65.31
28.05
f1 (ppm)

Compound 15

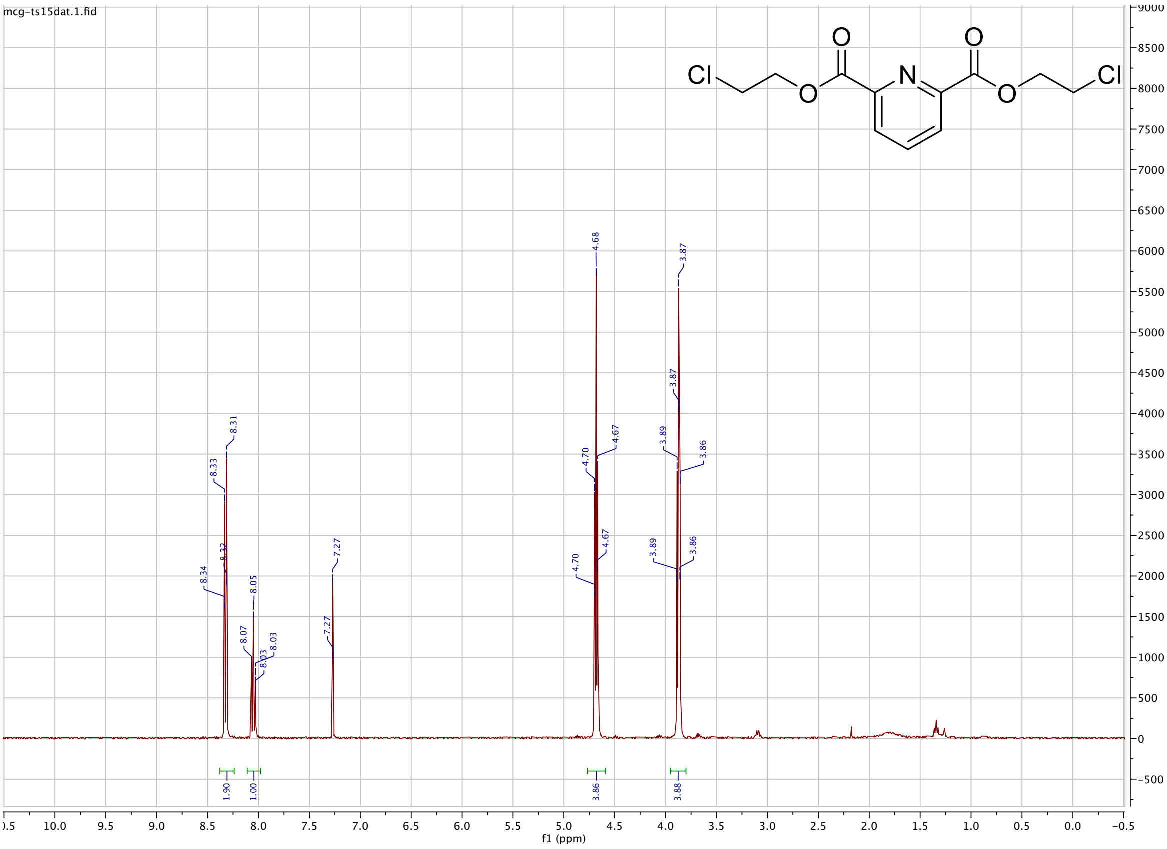
mcg-ts15dat.1.fid
f1 (ppm)

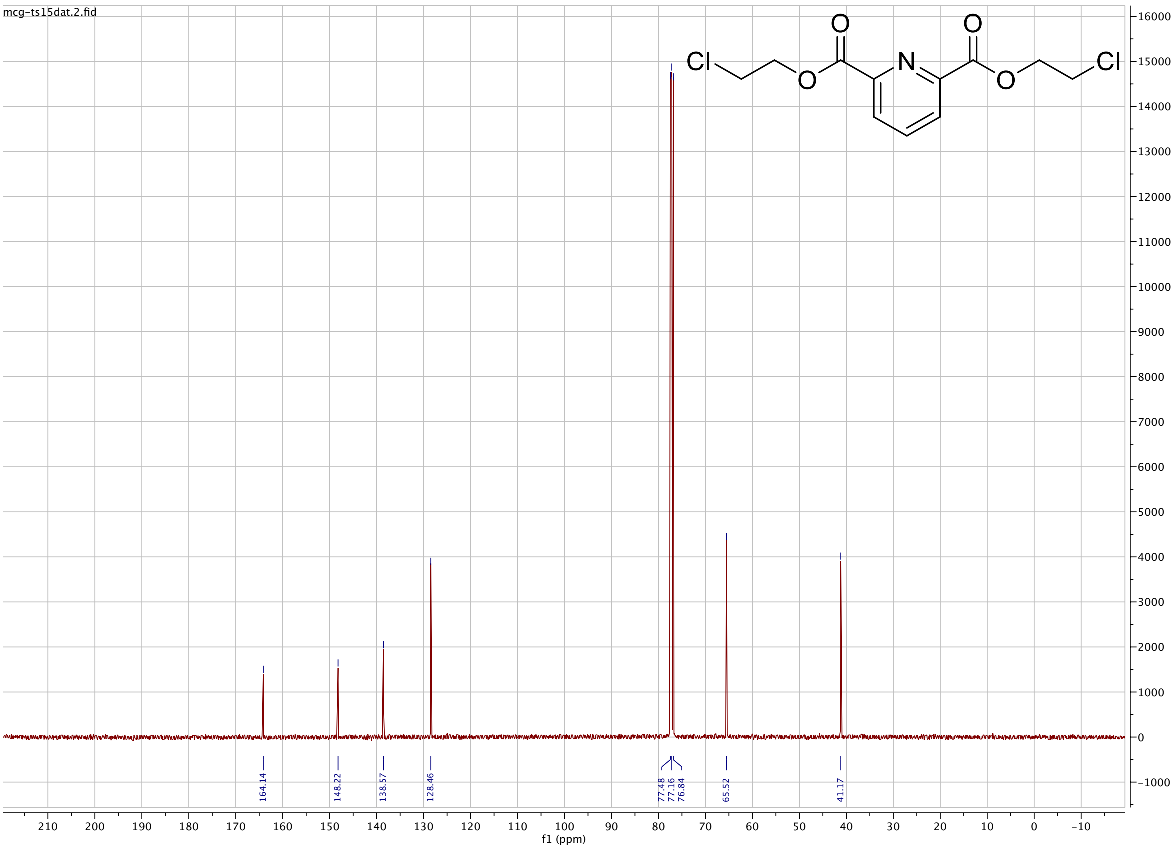
mcg-ts15dat.2.fid
164.14
148.22
138.57
128.46
77.48
77.16
76.84
65.52
41.17
f1 (ppm)

Compound 16

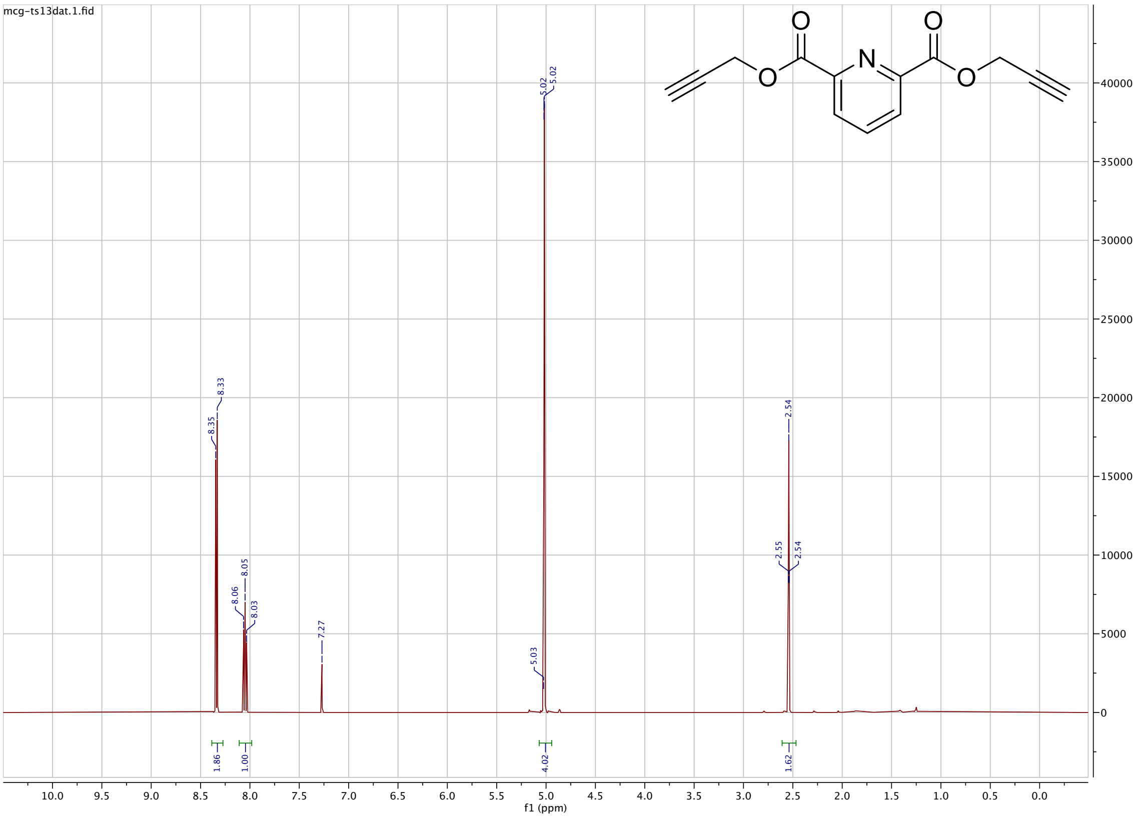
mcg-ts13dat.1.fid
8.35
8.33
8.06
8.05
8.03
7.27
5.02
5.02
5.03
2.55
2.54
2.54
1.86
1.00
4.02
1.62
O
O
N
O
O
f1 (ppm)

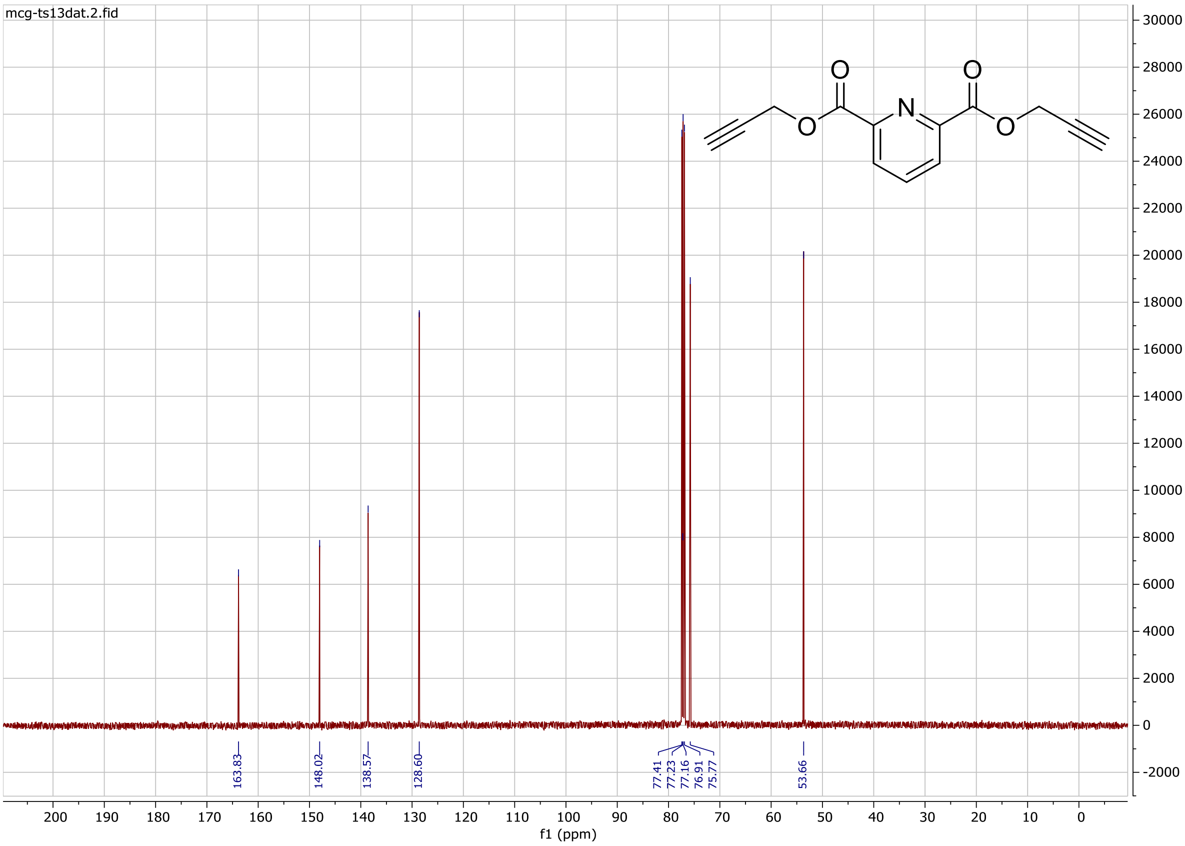
mcg-ts13dat.2.fid
163.83
148.02
138.57
128.60
77.41
77.23
77.16
76.91
75.77
53.66
O
O
N
O
O
f1 (ppm)

Compound 17

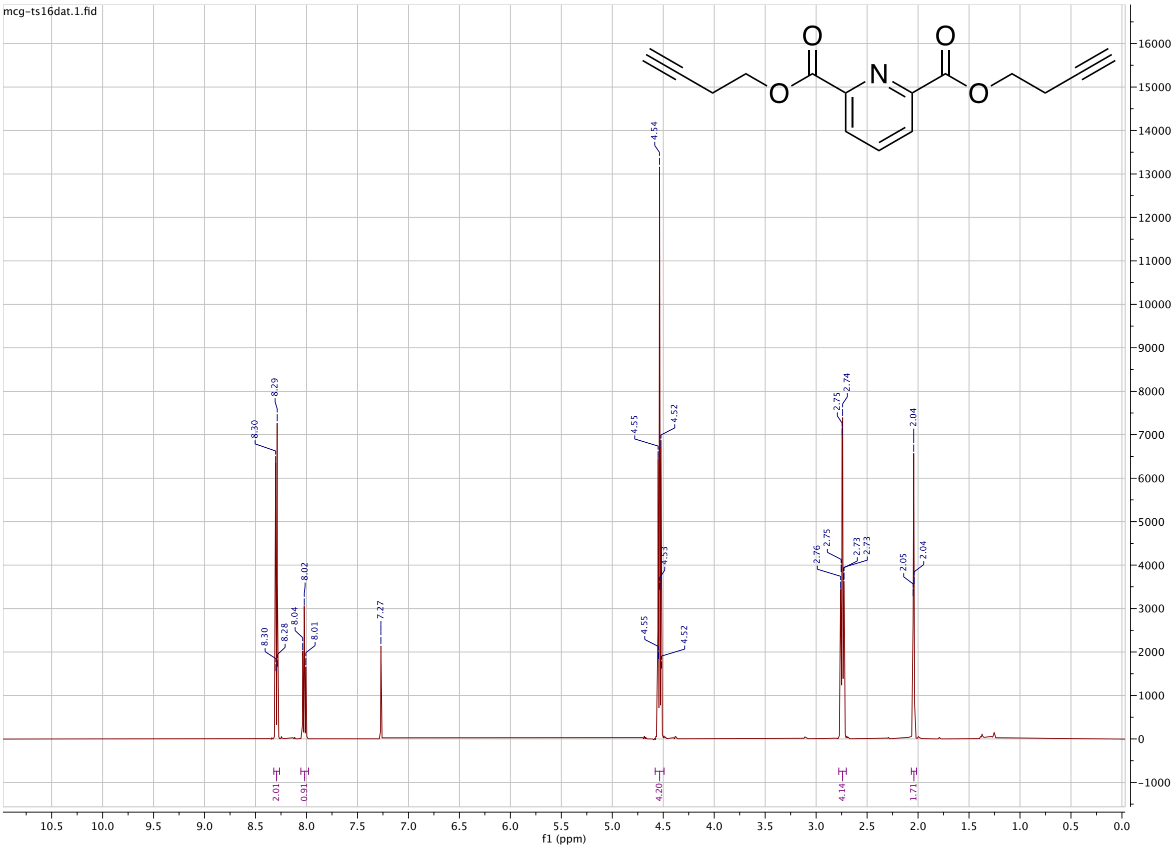

mcg-ts16dat.1.fid
O
O
N
O
O
8.30 8.29 8.30 8.28 8.04 8.02 8.01 7.27 4.54 4.55 4.52 4.53 4.55 4.52 2.75 2.74 2.76 2.75 2.73 2.73 2.04 2.05 2.04
2.01 0.91 4.20 4.14 1.71
10.5 10.0 9.5 9.0 8.5 8.0 7.5 7.0 6.5 6.0 5.5 5.0 4.5 4.0 3.5 3.0 2.5 2.0 1.5 1.0 0.5 0.0
f1 (ppm)
16000 15000 14000 13000 12000 11000 10000 9000 8000 7000 6000 5000 4000 3000 2000 1000 0 −1000

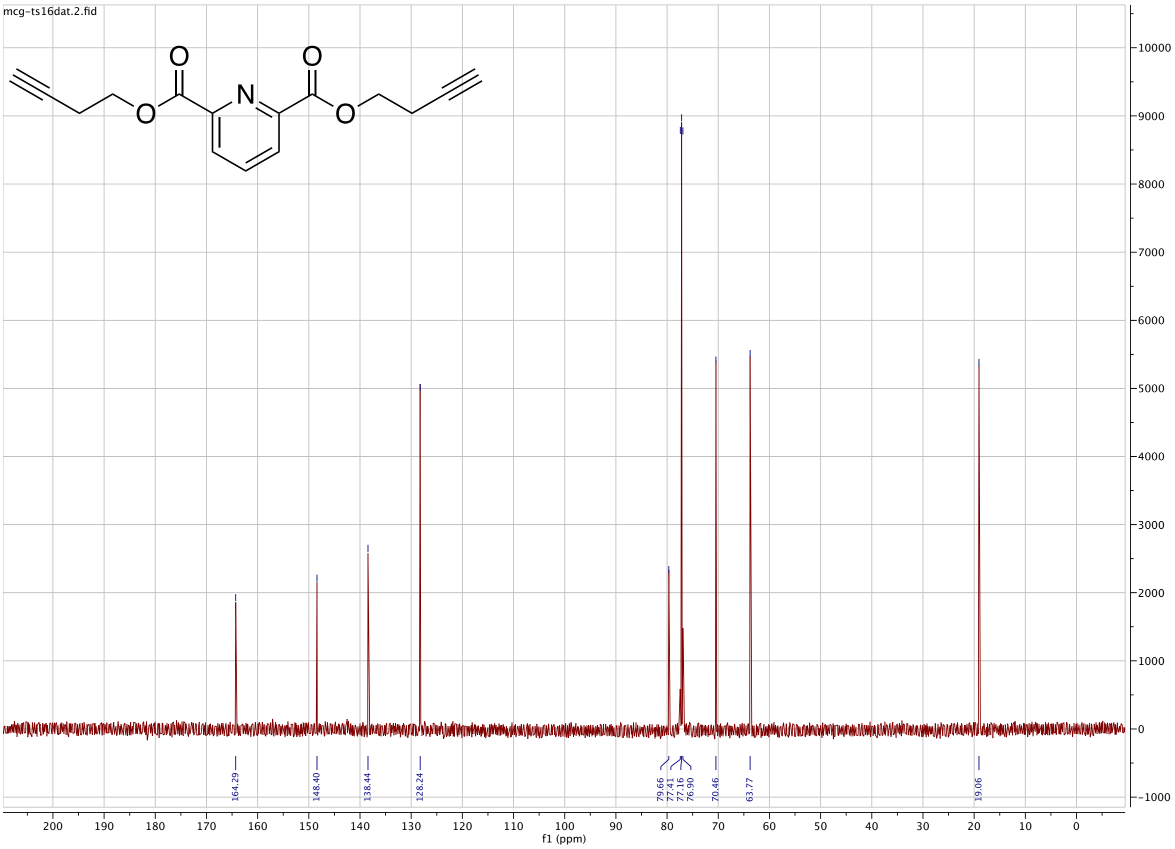

mcg-ts16dat.2.fid
O
O
N
O
O
164.29 148.40 138.44 128.24 79.66 77.41 77.16 76.90 70.46 63.77 19.06
200 190 180 170 160 150 140 130 120 110 100 90 80 70 60 50 40 30 20 10 0
f1 (ppm)
10000 9000 8000 7000 6000 5000 4000 3000 2000 1000 0 −1000

Compound 18

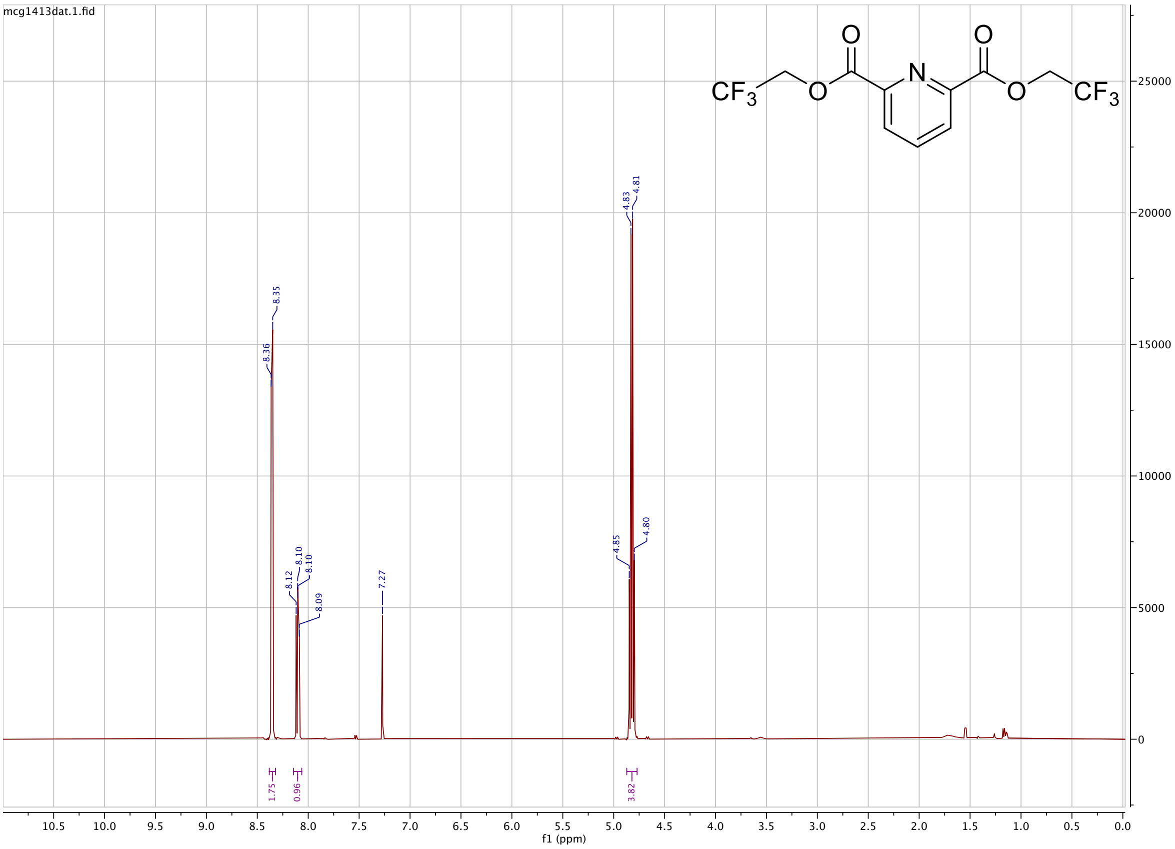

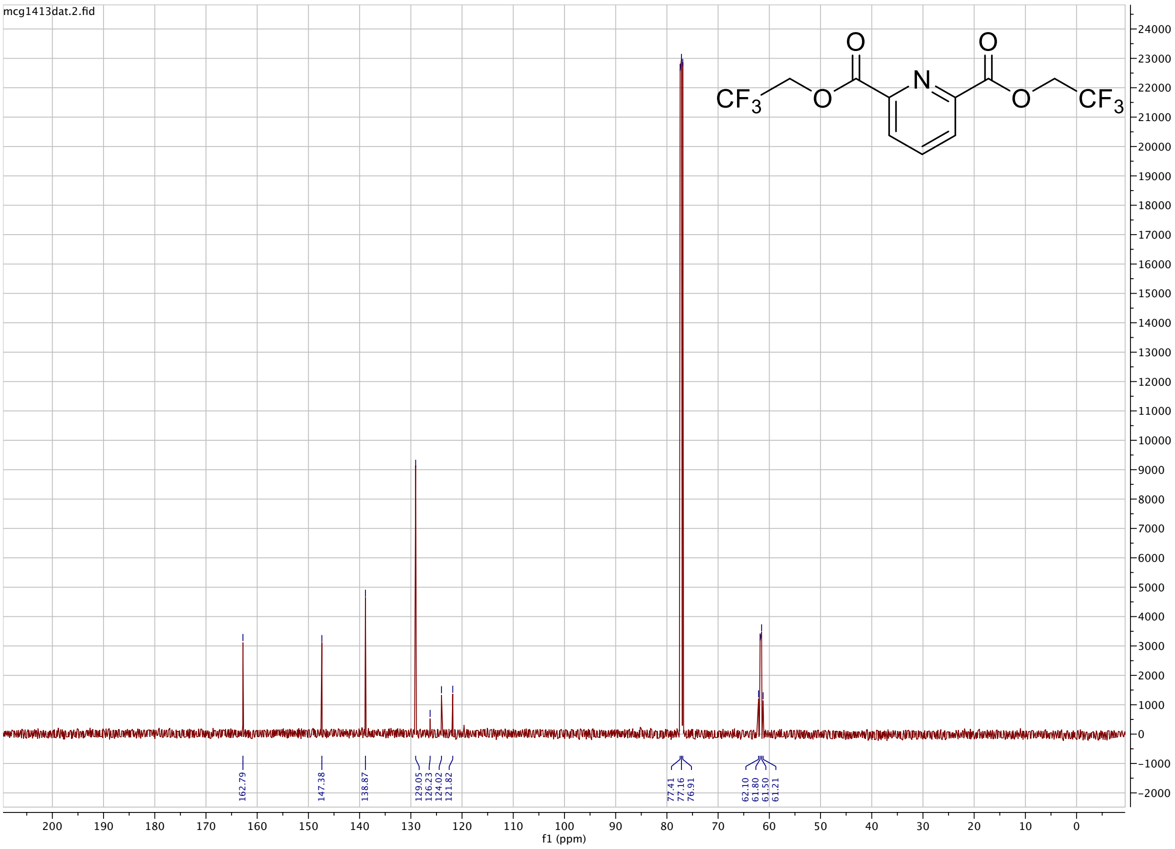

Compound 19

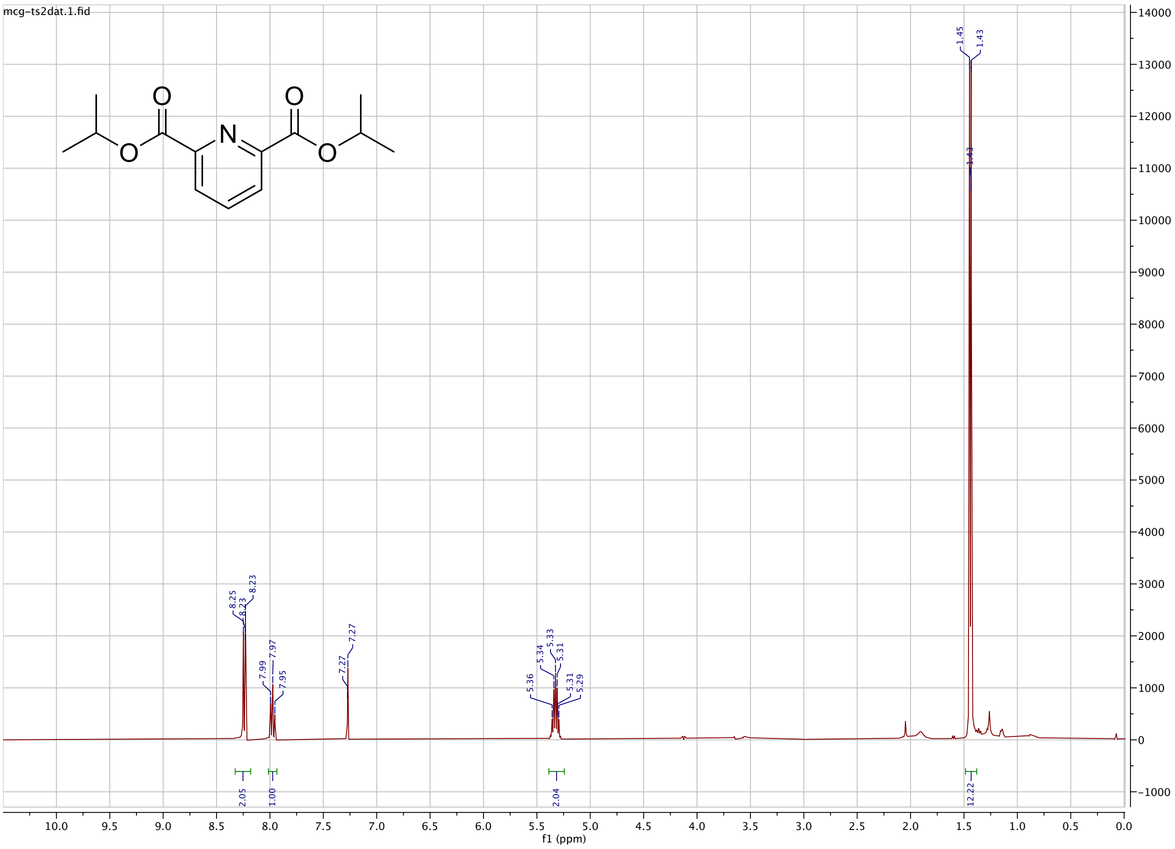
mcg-ts2dat.1.fid
O
N
O
O
O
8.25
8.23
8.23
7.99
7.97
7.95
7.27
7.27
5.36
5.34
5.33
5.31
5.31
5.29
1.45
1.43
1.43
2.05
1.00
2.04
12.22
f1 (ppm)

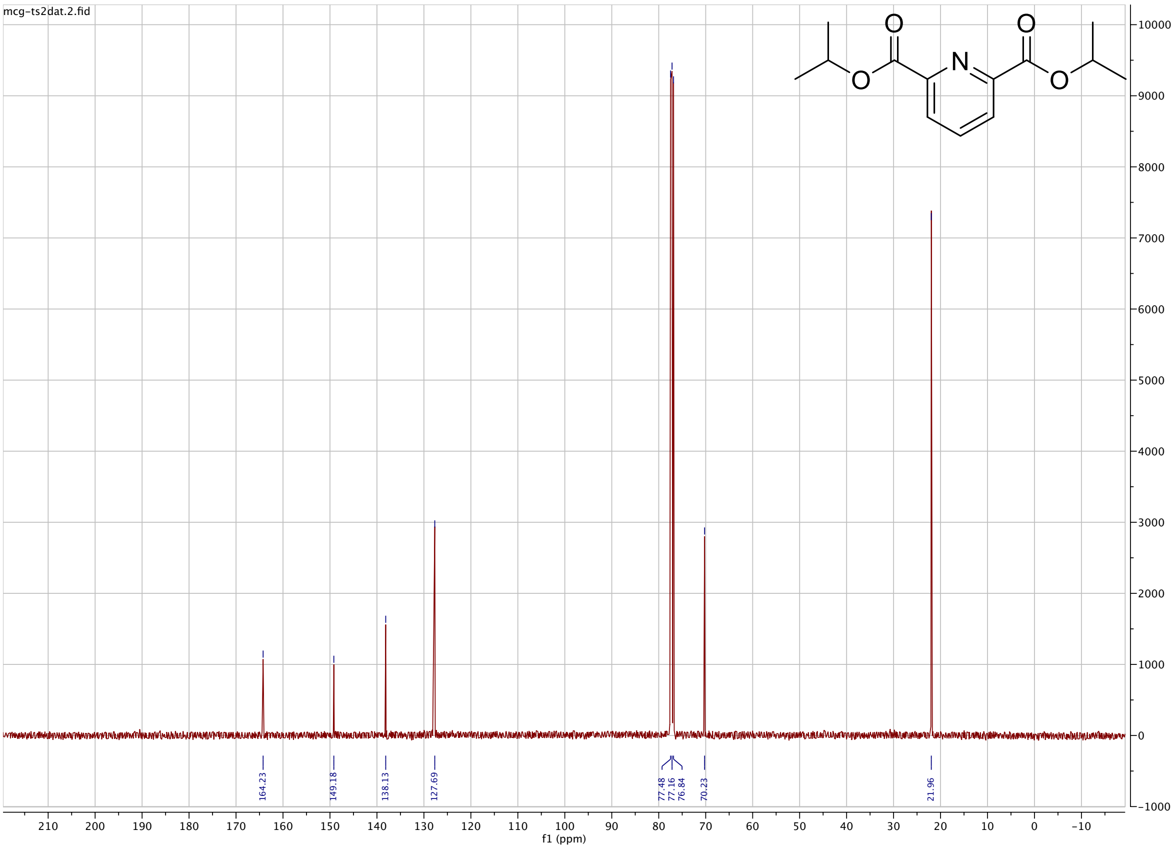
mcg-ts2dat.2.fid
O
N
O
O
O
164.23
149.18
138.13
127.69
77.48
77.16
76.84
70.23
21.96
f1 (ppm)

Compound 20

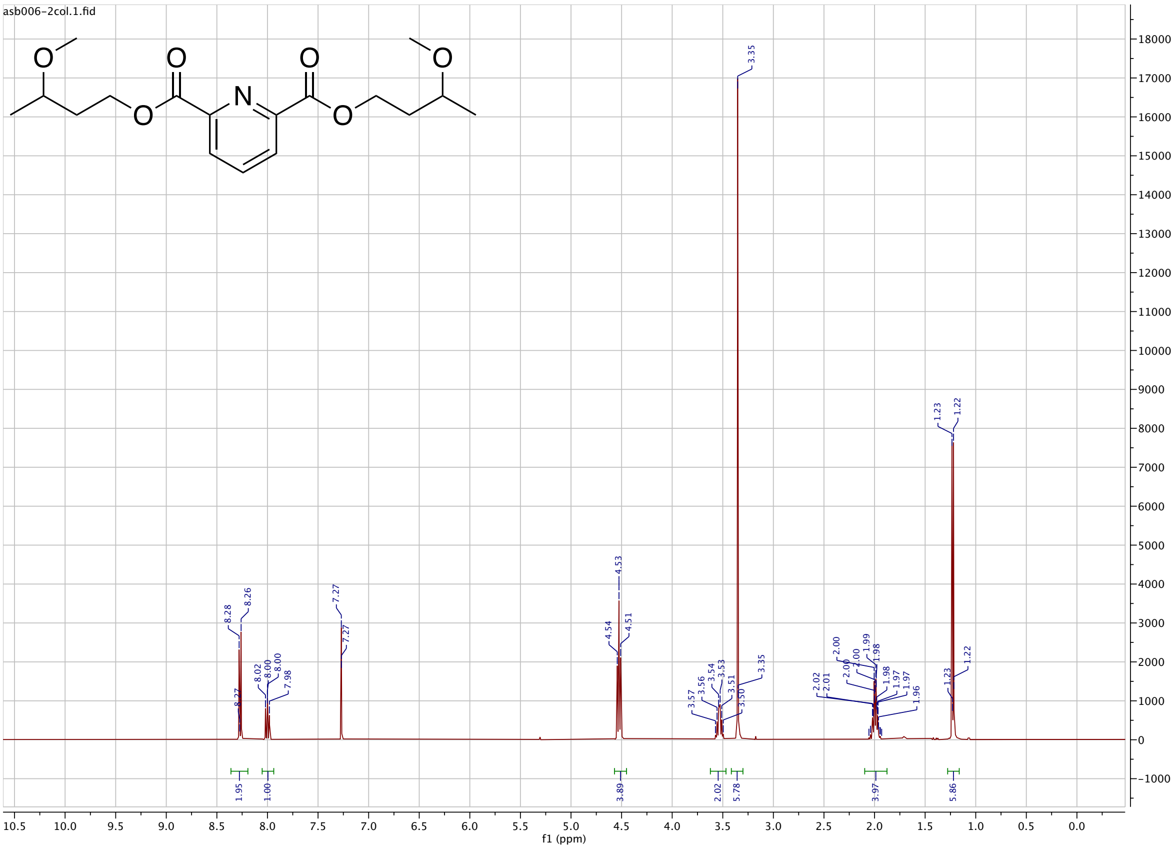
asb006-2col.1.fid
f1 (ppm)

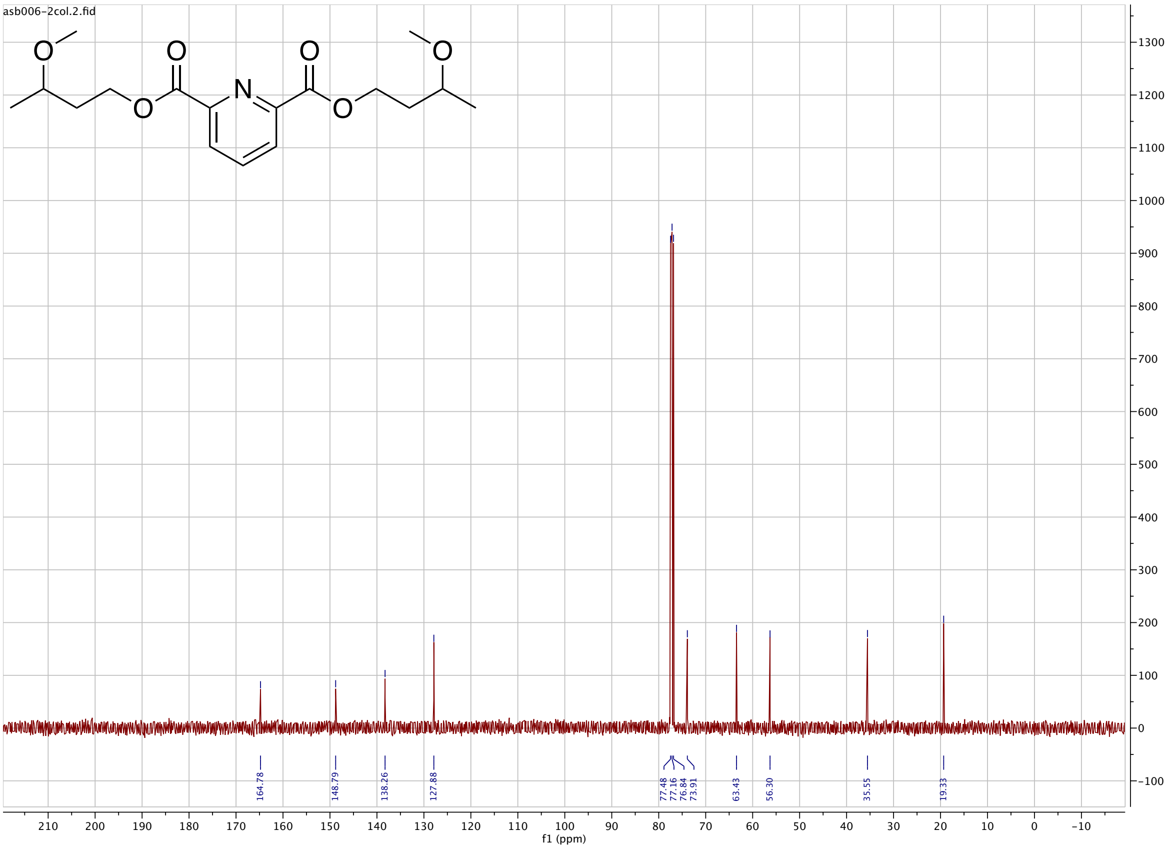
asb006-2col.2.fid
164.78
148.79
138.26
127.88
77.48
77.16
76.84
73.91
63.43
56.30
35.55
19.33
f1 (ppm)

Compound 21

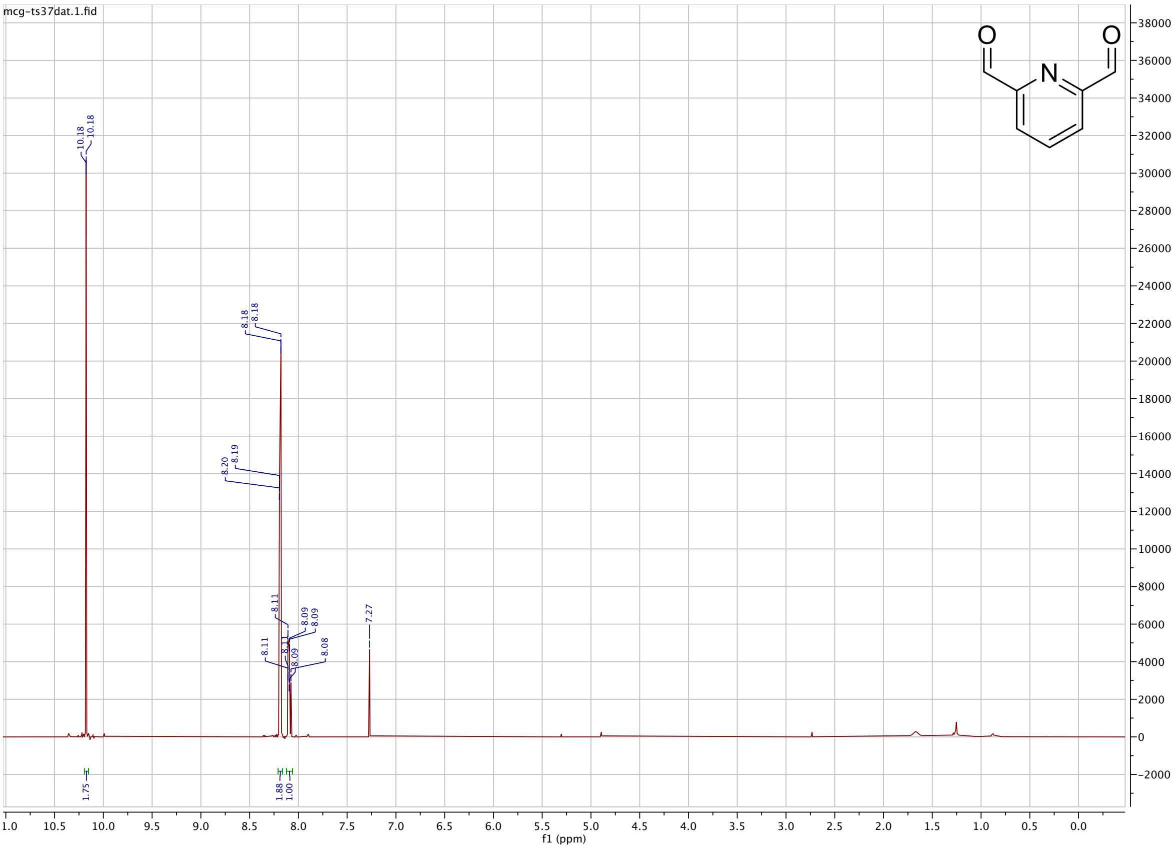
mcg-ts37dat.1.fid
10.18
10.18
8.18
8.18
8.20
8.19
8.11
8.11
8.11
8.09
8.09
8.09
8.08
7.27
1.75
1.88
1.00
f1 (ppm)

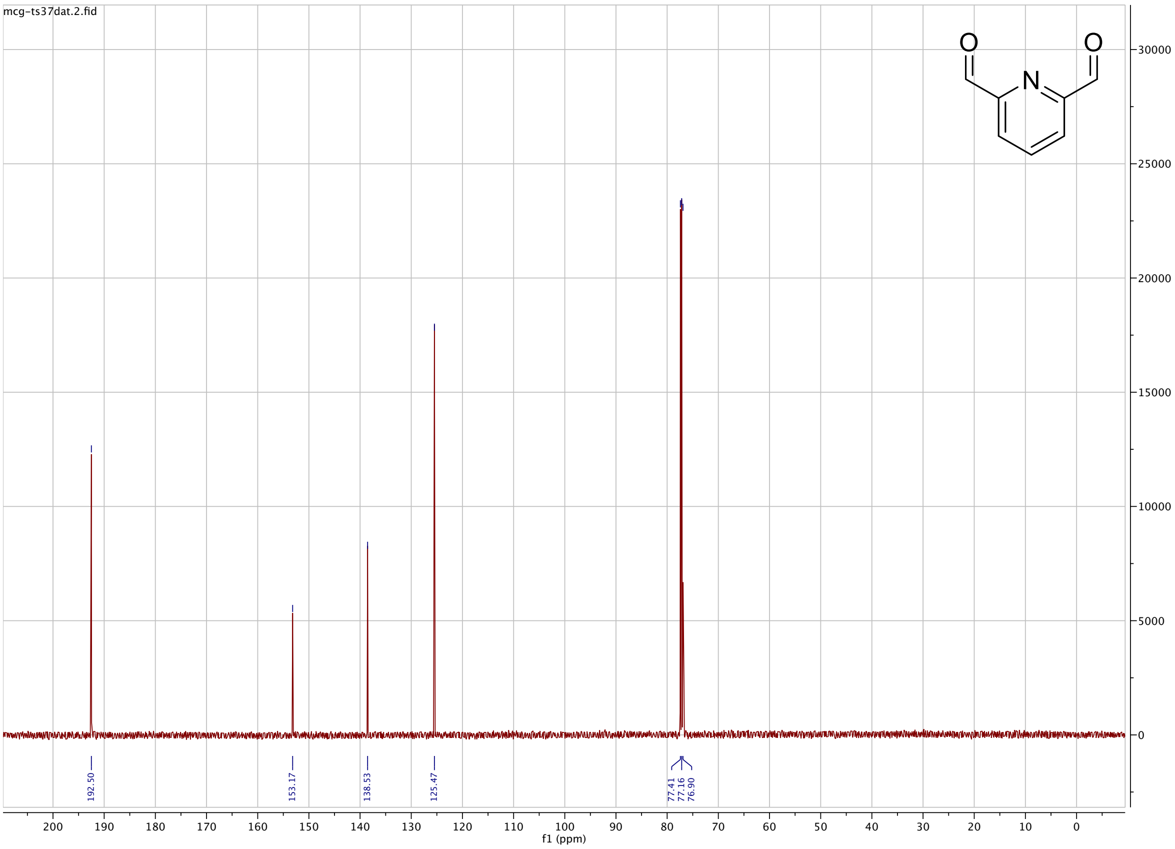
mcg-ts37dat.2.fid
192.50
153.17
138.53
125.47
77.41
77.16
76.90
f1 (ppm)
