# Supplementary material for: Repurposed inhibitor of bacterial dihydrodipicolinate reductase exhibits effective herbicidal activity
Source: Commun Biol. 2023 May 22;6:550. doi: 10.1038/s42003-023-04895-y (PMC10203105; doi:10.1038/s42003-023-04895-y)
Supplement: Supplementary file 2 — Description of Additional Supplementary Files [file 42003_2023_4895_MOESM2_ESM.pdf]

Description of Additional Supplementary Files

File name: Supplementary Data 1

Description: The source data used to generate figures 2, 5 and 7
